# Live imaging of marked chromosome regions reveals their dynamic resolution and compaction in mitosis

**DOI:** 10.1083/jcb.201807125

**Published:** 2019-03-11

**Authors:** John K. Eykelenboom, Marek Gierliński, Zuojun Yue, Nadia Hegarat, Hilary Pollard, Tatsuo Fukagawa, Helfrid Hochegger, Tomoyuki U. Tanaka

**Affiliations:** 1 Centre for Gene Regulation and Expression, School of Life Sciences, University of Dundee, Dundee, UK; 2 Data Analysis Group, School of Life Sciences, University of Dundee, Dundee, UK; 3 Genome Damage and Stability Centre, University of Sussex, Brighton, UK; 4 Graduate School of Frontier Biosciences, Osaka University, Suita, Osaka, Japan

## Abstract

When human cells enter mitosis, chromosomes undergo substantial changes in their organization to resolve sister chromatids and compact chromosomes. To comprehend the timing and coordination of these events, we need to evaluate the progression of both sister chromatid resolution and chromosome compaction in one assay. Here we achieved this by analyzing changes in configuration of marked chromosome regions over time, with high spatial and temporal resolution. This assay showed that sister chromatids cycle between nonresolved and partially resolved states with an interval of a few minutes during G2 phase before completing full resolution in prophase. Cohesins and WAPL antagonistically regulate sister chromatid resolution in late G2 and prophase while local enrichment of cohesin on chromosomes prevents precocious sister chromatid resolution. Moreover, our assay allowed quantitative evaluation of condensin II and I activities, which differentially promote sister chromatid resolution and chromosome compaction, respectively. Our assay reveals novel aspects of dynamics in mitotic chromosome resolution and compaction that were previously obscure in global chromosome assays.

## Abstract (precis)

Eykelenboom et al. track marked chromosome regions in live imaging of human cells with high spatial and temporal resolution to shed light on mitotic chromosome resolution and compaction dynamics.

## Introduction

At the beginning of mitosis, chromosomes undergo two major structural changes in metazoan cells. First, sister chromatids are resolved from each other along chromosome arms; this process involves removal of sister chromatid cohesion and elimination of topological DNA links (Nasmyth and Haering, 2009; Pommier et al., 2016; Uhlmann, 2016). Second, each sister chromatid is compacted; as a result, they become thicker in width and shorter in length (Hirano, 2016; Uhlmann, 2016). These two changes are a prerequisite for proper chromosome segregation toward opposite spindle poles during the subsequent anaphase. However, the precise timing and coordination of these two changes are still not fully understood.

Several factors regulate sister chromatid resolution and chromosome compaction. Sister chromatids are held together by the cohesin complex, which forms a ring structure consisting of SMC1, SMC3, RAD21, and SA1/2 (Nasmyth and Haering, 2009). For sister chromatid resolution, the cohesin complex must be removed along chromosome arms during prophase through the destabilizing activity of the WAPL (Wings apart-like protein homologue), while it is retained at the centromere to maintain sister chromatid cohesion until anaphase onset (Peters et al., 2008; Morales and Losada, 2018). In addition, topological DNA links (DNA catenation) from DNA supercoiling during DNA replication must also be removed by the de-catenation activity of topoisomerase II (topo II; Pommier et al., 2016; Piskadlo and Oliveira, 2017). Sister chromatid resolution starts in late G2 phase (Ono et al., 2013; Stanyte et al., 2018) and continues into prophase (Nagasaka et al., 2016). However, the dynamics of sister chromatid resolution in G2 and its regulation are not fully understood.

Furthermore, the condensin complex plays important roles in both sister chromatid resolution and chromosome compaction. The condensin complex exists as two forms—condensin I and II—that consist of the common SMC2 and SMC4 subunits and distinct non-SMC subunits such as NCAPD2 and NCAPD3 (for condensin I and II, respectively) (Hirano, 2012). Condensin I and II collaboratively generate helical arrays of nested chromatin loops (Gibcus et al., 2018; Walther et al., 2018). Moreover, condensin II operates earlier and contributes more to sister chromatid resolution than does condensin I (Ono et al., 2003; Shintomi and Hirano, 2011; Green et al., 2012; Hirano, 2012; Nagasaka et al., 2016). The precise timing of condensin I and II activity and their relative contribution to sister chromatid resolution and chromosome compaction remains to be fully elucidated.

The analysis of chromosome reorganization in early mitosis has been advanced by several new methods, which include chromosome conformation capture analyses (Hi-C; Naumova et al., 2013; Gibcus et al., 2018), differential visualization of sister chromatids (Nagasaka et al., 2016), and in vitro reconstitution of mitotic chromosomes (Shintomi et al., 2015). However, currently available methods cannot attain the following two goals. First, very few methods allow quantitative evaluation of sister chromatid resolution and chromosome compaction together. For example, Hi-C provides detailed information about chromosome compaction but not about sister chromatid resolution. A simultaneous evaluation of resolution and compaction is, however, critical since these processes might be coordinated. Second, although progression of global chromosome reorganization has been investigated in early mitosis, few studies analyzed regional chromosome reorganization in real time. Since global chromosome changes are the ensemble outcome of regional changes, such analyses could obscure dynamic regional changes of chromosomes—for example, any rapid or cyclical changes.

To achieve real-time measurements of regional chromosome dynamics, we investigated changes in specific chromosome regions over time in this study. Using bacteria-derived operator arrays (Michaelis et al., 1997; Belmont and Straight, 1998) we have created a fluorescence reporter system that quantitatively evaluates the timing of both sister chromatid resolution and chromosome compaction at chosen chromosome regions in human cells. This has allowed us to study dynamic chromosome reorganization from G2 phase to early mitosis by live cell microscopy.

## Results

### Visualizing sister chromatid resolution and compaction at a chosen region in live human cells

**Figure 1.**
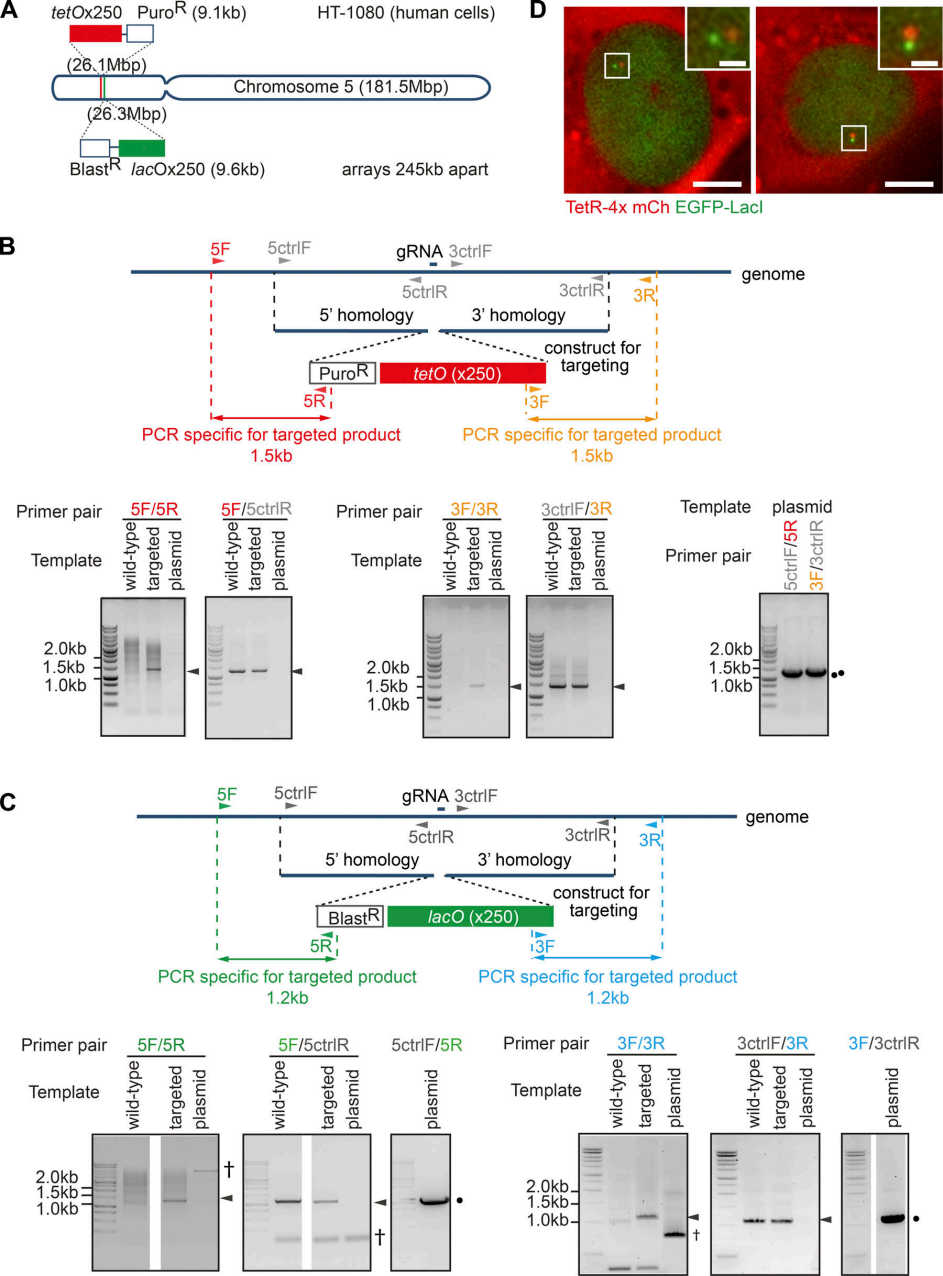
**Targeting *tet* and *lac* operator arrays to a selected chromosome region. (A)** Diagram depicting the location of *tetO* and *lacO* introduced into human chromosome 5. **(B and C)** The maps (top) show the DNA construct used for targeted integration of *tetO* and *lacO* and the corresponding positions on the genome of the guide RNA (gRNA for CRISPR-Cas9) and PCR primers (e.g., 5F, 5R, etc.) for integration check. The DNA electrophoreses (bottom) show the results of PCR using template DNA from the WT genome, the genome with targeted integration (targeted), and, as control, the purified plasmid targeting construct. Arrowheads and black dots indicate PCR products expected from correct integration and the purified plasmid, respectively. Nonspecific bands are indicated by a single dagger (†). Results demonstrate correct targeted integration of *tetO* and *lacO*. **(D)** Visualization of these arrays by expression of TetR-4x mCherry and EGFP-LacI in HT-1080 cells (TT75). The white square boxes represent the zoomed region shown in the upper right-hand corners. Scale bars, 10 µm (main) and 1 µm (zoomed inset).

To analyze mitotic chromosome reorganization, we developed an assay system in live HT-1080 diploid human cells. Using CRISPR-Cas9 technology we integrated a *tet* operator array and a *lac* operator array (Lau et al., 2003) with a 250-kbp interval to a region of chromosome 5 with low gene density (Fig. 1, A–C). The *tet* and *lac* operators (*tetO* and *lacO*) were bound by Tet-repressor fused to four monomer-Cherry fluorescent proteins (TetR-4xmCh) and by the Lac-repressor fused to GFP and a nuclear localization signal (EGFP-LacI-NLS), thus visualized as red and green fluorescent dots, respectively (Fig. 1 D). We chose a cell line where red and green fluorescent dots were found in proximity, reasoning that, in this cell line, *tetO* and *lacO* were integrated on the same copy of chromosome 5. The *tetO* and *lacO* were stably maintained during cell proliferation since their signal intensity did not become weakened. As implied previously (Chubb et al., 2002; Thomson et al., 2004), integration of these operators onto chromosome 5 did not affect cell cycle progression or the fidelity of chromosome segregation; indeed, there was no change in DNA content of these cells as determined by flow cytometry or no missegregation of chromosome 5 observed by microscopy.

**Figure 2.**
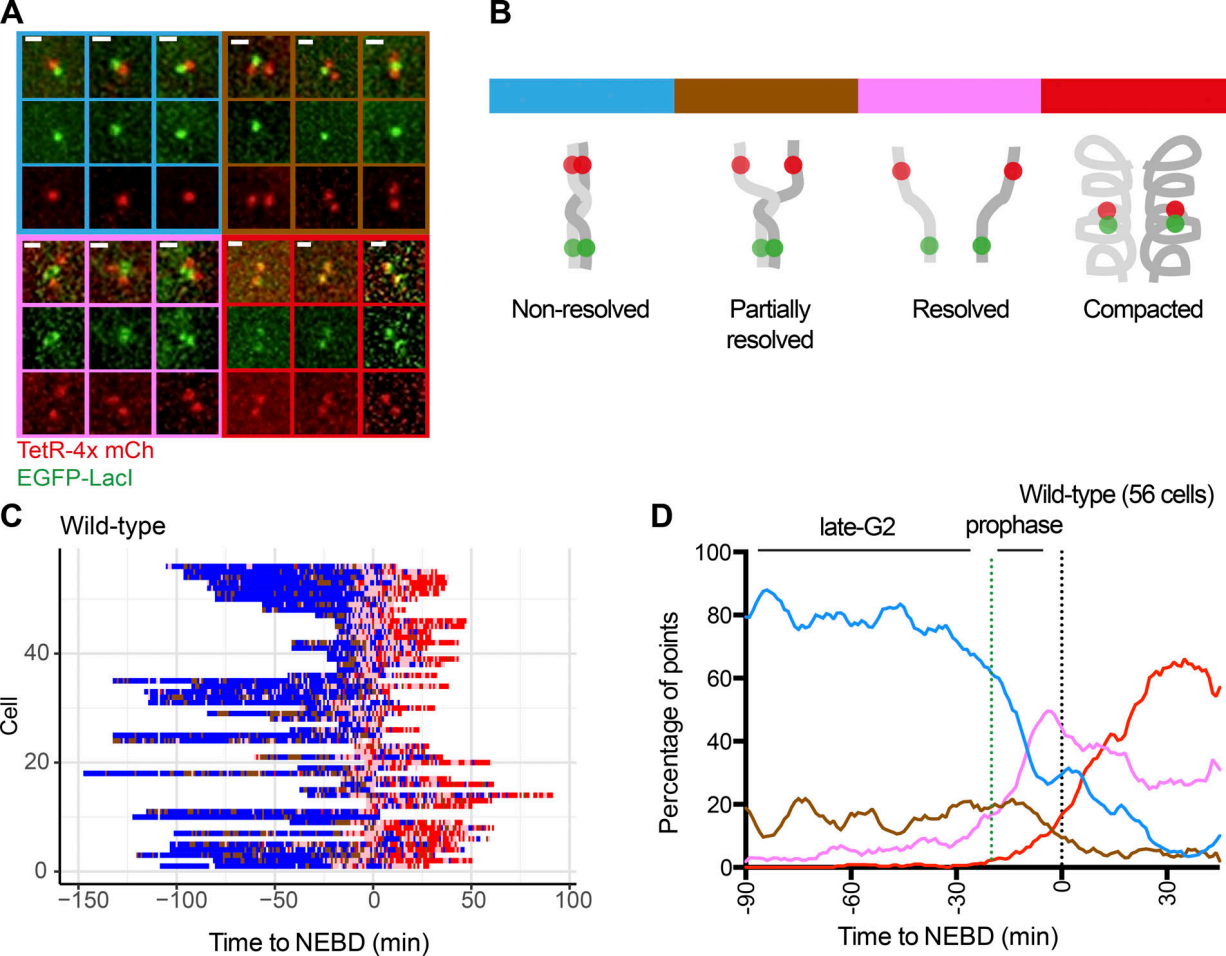
**A fluorescence reporter for observing configuration of a selected chromosome region in live cells. (A)** Representative images of the configurations of the fluorescence reporter observed in TT75 cells (see Fig. 1). Designated color codes for each configuration are indicated in the image frames. Scale bars, 1 µm. **(B)** Diagram shows the reporter configurations in A, with the same color codes as in A. **(C)** Change in the configuration of the fluorescence reporter over time (*x* axis) as observed in individual live cells (across the *y* axis). TT75 cells were synchronized by a double-thymidine block and released, images were taken every minute, and, at each time point, the configuration of the reporter was determined as in A and B (shown with the same color codes). Data from individual cells were aligned relative to NEBD (defined as time zero). **(D)** The proportion of each configuration (color-coded as in A and B) was determined from the data in C and plotted over time with smoothing (across 9 min). The number of analyzed cells at each time point is between 15 and 55 (mean: 33).

We acquired microscopy images of the above cells and analyzed the z-stack images in 3D space, which revealed various configurations of the fluorescent dots (Fig. S1 A). During interphase, we observed one green dot and one red dot (Fig. 2 A; defined as blue state). By contrast, in early mitosis (shortly before and after nuclear envelope breakdown [NEBD]; see below), we often observed (a) one green dot and two red dots (or vice versa; defined as brown state), (b) two green dots and two red dots without colocalization (defined as pink state), and (c) two green dots and two red dots with colocalization of each green and red dot (defined as red state; Fig. 2 A). These states are likely to reflect chromosome reorganization during early mitosis, as follows (Fig. 2 B): (a) the blue state represents “nonresolved” sister chromatids (if cells have progressed through S phase), (b) the brown state reflects “partially resolved” sister chromatids, (c) the pink state shows “resolved” (but not compacted) sister chromatids, and (d) the red state indicates resolved and “compacted” chromatids. Notably, the red “compacted” state was found to be the most frequent state in the final minutes before anaphase onset, suggesting that it reflects the “end” metaphase chromosome structure.

To analyze chromosome reorganization in early mitosis, we released cells with the fluorescent dots from a double thymidine block and acquired live-cell images every minute between 8 and 12 h (relative to the release). We were able to identify the timing of NEBD in individual cells, as it caused dispersion of the EGFP-LacI-NLS signal (the fraction not bound to *lac* operators) from the nucleus (Fig. S1 B). For individual cells, we aligned the sequence of the states of fluorescent dots (as defined in Fig. 2, A and B) relative to NEBD (defined as time zero; Fig. 2 C). Then, we plotted the proportion of cells displaying each state against time (Fig. 2 D). We noticed that as cells approached NEBD, the pink “resolved” state increased its frequency. After NEBD, there was an increase in occurrence of the red “compacted” state. These observations suggest that chromosome reorganization proceeds from sister chromatid resolution to chromosome compaction, as assumed in Fig. 2 B. This conclusion was also supported by measurement of the distances between fluorescent dots (Fig. S1, C–F): that is, the mean distances between sister *tetO*s and between sister *lacO*s increased before NEBD, reflecting sister chromatid resolution (Fig. S1 D), while the mean distance between *tetO* and *lacO* gradually decreased after NEBD, reflecting chromosome compaction (Fig. S1, E and F).

To analyze the dynamics of a marked chromosome region in different cells, we inserted *tetO* and *lacO* with a 100-kbp interval on the Z chromosome of DT40 cells (Fig. S2, A and B) and visualized them using the same method as above. In these cells, we could identify the same four configurations of the fluorescent dots as above (Fig. S2 C). The pink “resolved” and red “compacted” states appeared with similar timing to that observed in human cells (Fig. S2 D). We conclude that a marked chromosome region behaves similarly during early mitosis in different vertebrate species and in different chromosome contexts.

### Sister chromatids cycle between nonresolved and partially resolved states with an interval of a few minutes during late G2 phase before attaining full resolution in prophase

**Figure 3.**
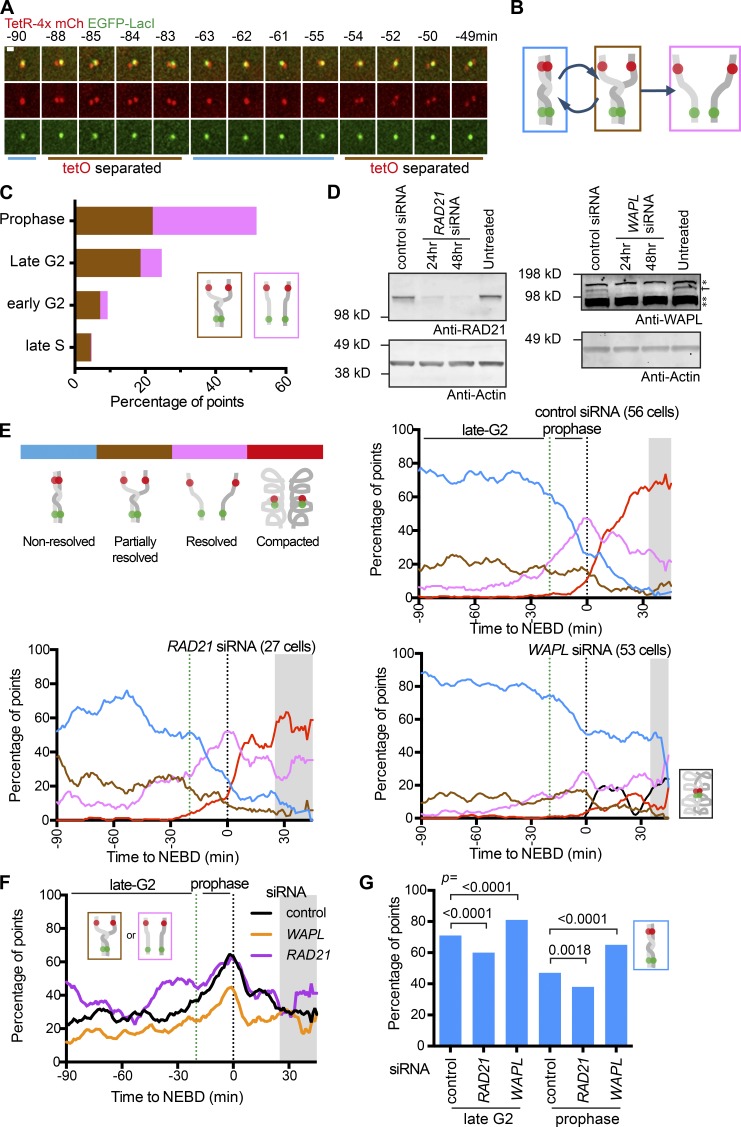
**Sister chromatids cycle between nonresolved and partially resolved states during late G2 phase, antagonistically regulated by cohesins and WAPL. (A)** Time-sequence images of the fluorescence reporter showing cyclical separation of sister *tetO*. Times are relative to NEBD. Scale bar, 1 µm. **(B)** Diagram depicting cyclical partial sister resolution (brown state) in late G2 phase, leading to full resolution (pink state) in prophase. **(C)** The proportion of the brown “partially resolved” or the pink “resolved” state during the indicated cell cycle phases. The cell cycle phases were determined in TT104 cells carrying Cerulean-PCNA (*n* = 61 cells in late S and early G2 phase and 26 cells in late G2 and prophase). **(D)** Western blots for RAD21 (left) or WAPL (right) proteins from cells after siRNA treatment for the indicated time. The control siRNA treatment was over 48 h. Actin is shown as a loading control. Asterisks indicate nonspecific binding of the anti-WAPL antibody. **(E)** The proportion of each configuration in cells treated with control, RAD21, and WAPL siRNA. Color codes are as in diagram (top, left). TT75 cells were arrested by a double-thymidine block and released and also treated with siRNA, as in Fig. S2 I. WAPL siRNA led to colocalization of all four fluorescent dots (black line) after NEBD in some cells. Data from individual cells are shown in Fig. S2 K. The number of analyzed cells at each point was between 10 and 53 (mean: 33) for control; 10 and 27 (mean: 19) for RAD21; 10 and 52 (mean: 36) for WAPL siRNA, except for the gray-shaded areas where <10 cells were analyzed. **(F)** The proportion of the brown “partially resolved” plus pink “resolved” state after control, RAD21 and WAPL siRNA treatment. The data were taken from E. The gray shaded area indicates time points that include <10 cells for at least one siRNA treatment. **(G)** Graph shows the proportion of the blue “nonresolved” state for control, RAD21, and WAPL siRNA during late G2 phase or prophase. These data were taken from E. P values were obtained using a chi-square test. *n* = 520–3,081 time points.

Further analysis of the HT-1080 cells revealed that the brown “partially resolved” state often (∼20% of time points) appeared up to 2 h before NEBD (Fig. 2, C and D). The brown state typically appeared and continued for a few minutes before returning to the blue “nonresolved” state (Fig. 3 A). Thus, the blue and brown states show cyclical exchanges before being converted to the pink “resolved” state (Fig. 3 B). To frame the timing of this process in the cell cycle, we defined S, G2, and prophase in our real-time imaging, as follows: We identified S phase cells by visualizing a component of the replication machinery, proliferating cell nuclear antigen (PCNA) tagged with mCerulean. Since Cerulean-PCNA shows characteristic globular signals during S phase (Fig. S2 E; Kitamura et al., 2006; Thomson et al., 2010), we defined the end of S phase as the time its globular signals disappeared (Fig. S2 F, left). Our observations of PCNA and NEBD by live-cell imaging suggested that the length of G2 phase (between the end of S phase and the start of prophase) was 5–7 h (Fig. S2 F, right), which is consistent with other studies (Defendi and Manson, 1963). We also defined prophase as a 20-min time window before NEBD, according to previous estimates (Liang et al., 2015) and based on the global change in the chromosome volume observed in our cells (Fig. S2 G).

The brown “partially resolved” state appeared infrequently in late S phase (the last 30 min of S phase) and early G2 phase (first 90 min of G2 phase), but its frequency increased in late G2 (last 120 min of G2 phase; Fig. 3 C and Fig. S2 H). Subsequently, in prophase, the pink “resolved” state increased in frequency (Fig. 3 C). The brown state was also observed in ≤20% of DT40 cells in late G2 phase (Fig. S2, C and D). We conclude that sister chromatid resolution begins in late G2 phase and completes during prophase, at least at the chromosome region of our study. Our results are consistent with other reports that sister chromatid resolution begins before mitosis (Ono et al., 2013; Stanyte et al., 2018) and continues in prophase (Nagasaka et al., 2016). As suggested by Stanyte et al. (2018), sister chromatids cycle between nonresolved and partially resolved states in late G2 phase. We found that the two states interchange with a period of a few minutes (i.e., more dynamically than previously thought).

### Sister chromatid resolution is antagonistically regulated by cohesins and WAPL, not only during prophase but also in G2 phase

Previous studies suggested that maintenance of catenated DNA requires cohesins (Farcas et al., 2011; Sen et al., 2016) and cohesin removal from chromosome arms by WAPL promotes sister chromatid resolution in prophase (Peters et al., 2008). We tested if these conclusions are reproduced with our assay. We also addressed whether the initial sister chromatid resolution in late G2 phase, described above, depends on cohesin removal by WAPL. We used siRNA to deplete the cohesin subunit RAD21 or WAPL within 24–48 h of transfection (see Fig. 3 D and Fig. S2 I). By observing fixed metaphase chromosomes, we found that sister chromatids were morphologically less distinct after WAPL depletion, confirming the previously observed global defect in sister chromatid resolution caused by WAPL siRNA (Gandhi et al., 2006; Fig. S2 J).

We then scored how fluorescent dots changed their configuration from late G2 to prometaphase after 48 h of siRNA treatment (Fig. 3 E and Fig. S2, K and L). Relative to control siRNA, depletion of RAD21 caused (a) an increase in the brown “partially resolved” and pink “resolved” states (Fig. 3 F) and, conversely, (b) a decrease in the blue “nonresolved” state (Fig. 3 G) in late G2 and prophase. In contrast, depletion of WAPL led to the opposite outcomes in late G2 and prophase. Relatively modest changes with RAD21 siRNA may be due to residual RAD21; consistently, even a small amount (∼20%) of cohesins is sufficient to maintain sister chromatid cohesion (Carvalhal et al., 2018). We conclude that cohesins inhibit precocious sister chromatid resolution in late G2 and prophase while WAPL promotes sister chromatid resolution during these phases. Thus, cohesins and WAPL play antagonistic roles in sister chromatid resolution not only during prophase but also during late G2 phase.

### Local cohesin enrichment regionally prevents precocious sister chromatid resolution during prophase

**Figure 4.**
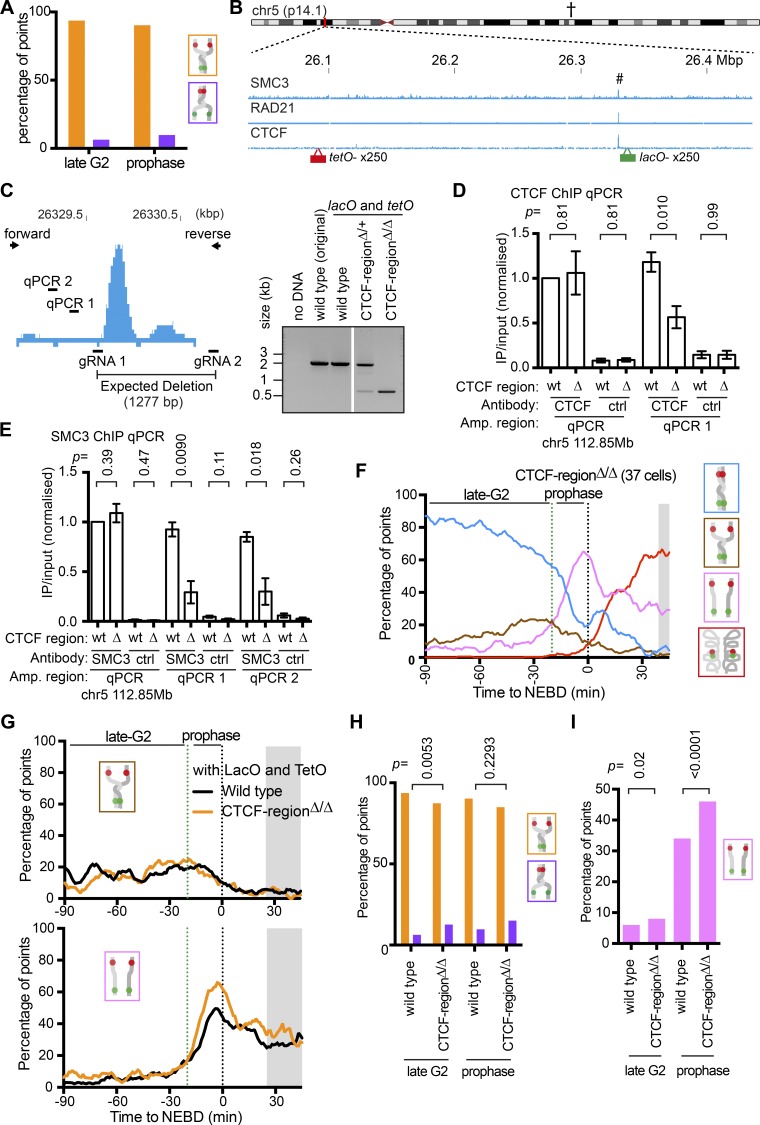
**Local reduction of cohesins at their enrichment site leads to precocious sister chromatid resolution in that region during prophase. (A)** Graph shows the proportion of *tetO* (orange) and *lacO* (purple) sister separation among all “partially resolved” states in WT cells (TT75) during late G2 phase or prophase. *n* = 175–381 time points. **(B)** The ChIP-seq data show the distribution of SMC3, RAD21, and CTCF along the genomic region around integration sites of *lacO* and *tetO*. The ChIP-seq data are taken from published data (ENCODE Project Consortium, 2012). **(C)** The map (left) is a zoomed view of the CTCF ChIP-seq peak at the chromosome region highlighted with # in B. The map also shows the positions on the genome to which guide RNA (gRNA; for CRISPR-Cas9) and PCR primers (forward and reverse, to confirm deletion) correspond, as well as the genome intervals (qPCR1 and qPCR2) for ChIP-qPCR in D and E. PCR (right) gave a band of the expected size, ∼2 kb from intact genome DNA and ∼0.7 kb from genomic DNA containing the deletion. **(D and E)** Graphs show results of ChIP-qPCR. An antibody against CTCF or SMC3, or a nonspecific antibody (mouse or rabbit IgG), was used for ChIP with WT (wt) or CTCF-region^Δ/Δ^ (Δ) cells (TT75 and TT108, respectively). The genome intervals (qPCR1 and qPCR2 in C) were amplified by PCR following ChIP. A region on chromosome 5 (112.85 Mbp), where CTCF and cohesins are enriched, was also amplified by PCR as a control (indicated as † in B and zoomed in Fig. S3 D). For each sample, the yield (IP/input DNA) was normalized to that at the control region in WT cells. ChIP-qPCR was repeated four and three times for CTCF and SMC3, respectively (Fig. S3, E and F), and means and standard errors are shown in graphs. P values were obtained by *t* tests. **(F)** The proportion of each configuration of the fluorescence reporter for CTCF-region^Δ/Δ^ cells (TT108) was plotted over time. TT108 cells were synchronized and analyzed as in Fig. 2, C and D. Data from individual cells are shown in Fig. S3 G. The number of analyzed cells at each point was between 10 and 36 (mean: 26), except for the gray-shaded area where <10 cells were analyzed. **(G)** The proportion of the brown “partially resolved” (top) or pink “resolved” (bottom) state for WT or CTCF-region^Δ/Δ^ cells. These data were taken from Figs. 2 D and 4 F. The gray-shaded area is as in Fig. 3 F. **(H)** The graph shows the proportion of *tetO* (orange bars) and *lacO* (purple bars) sister separation among all “partial resolution” states in WT and CTCF-region^Δ/Δ^ cells during late G2 phase or prophase. P values were obtained using the chi-square test. *n* = 93–381 time points. **(I)** The proportion of the pink “resolved” state for WT or CTCF-region^Δ/Δ^ cells during late G2 phase or prophase. These data were taken from Figs. 2 D and 4 F. P values were obtained using the chi-square test. *n* = 680–2,406 time points. ctrl, control; IP, immunoprecipitated.

In the brown “partially resolved” state of fluorescent dots (see Fig. 2, A and B), *tetO* (red dot) showed much more frequent sister separation than *lacO* (green dot) in late G2 and prophase (Fig. 4 A). It is unlikely such sister *tetO* separation was an artifact of this array, since the *lacO* also showed more frequent sister separation (than in the original cell line) when it was integrated at another chromosome region in another cell line (Fig. S3, A and B). In the original cell line, sister *lacO* separation may be infrequent because of a specific chromosome feature. We inspected the genomic region where the operator arrays were integrated using publicly available chromatin immunoprecipitation (ChIP; followed by DNA sequencing [ChIP-seq]) datasets (ENCODE Project Consortium, 2012). The *lacO* was located within 5 kbp of a cohesin-enriched region (Fig. 4 B; SMC3 and RAD21). This close proximity to a cohesin-enriched site might contribute to the reduced (or delayed) *lacO* separation. This cohesin peak coincides with a CTCF (CCCTC-binding factor)-enriched region (Fig. 4 B). CTCF is a protein that binds specific DNA sequences and acts as a barrier to cohesin movement leading to its local accumulation (Parelho et al., 2008; Wendt et al., 2008). Therefore, deletion of the CTCF binding sequence of this region might reduce the level of cohesins found there.

To investigate the outcome of a reduced cohesin level at this region, we deleted a 1277-bp DNA sequence corresponding to the CTCF enrichment site containing three CTCF-binding consensus sites (Ziebarth et al., 2013) using CRISPR-Cas9 technique (Fig. 4 C, left). Deletion of this region was confirmed by PCR and DNA sequencing on the two homologous chromosomes (Fig. 4 C, right; and Fig S3 C) and was designated as “CTCF-region^∆/∆^.” The level of chromosome-bound CTCF or SMC3 was examined in the vicinity of this region by ChIP followed by quantitative PCR (ChIP-qPCR; Fig. 4 C, left; and Fig. S3 D). This confirmed that deletion of the CTCF binding sites resulted in reduction of chromosome-bound CTCF and SMC3 by 52% and 67%, respectively (Fig. 4, D and E; and Fig. S3, E and F).

In the CTCF-region^∆/∆^ strain, we scored how fluorescent dots changed their configuration from late G2 to prometaphase (Fig. 4 F and Fig. S3, G and H). In the CTCF-region^∆/∆^ cell line, the overall fraction of the brown “partially resolved” state was similar to the WT control (Fig. 4 G, top), but there was a slight increase in sister separation of the *lacO* (green fluorescent dot) in late G2 phase (Fig. 4 H). Moreover, the CTCF-region^∆/∆^ cell line showed an earlier and greater increase in the pink “resolved” state during prophase, compared with WT (Fig. 4, G [bottom] and I). We conclude that a local reduction in the cohesin level leads to precocious sister chromatid resolution there during prophase, which is presumably due to weaker sister chromatid cohesion.

**Figure 5.**
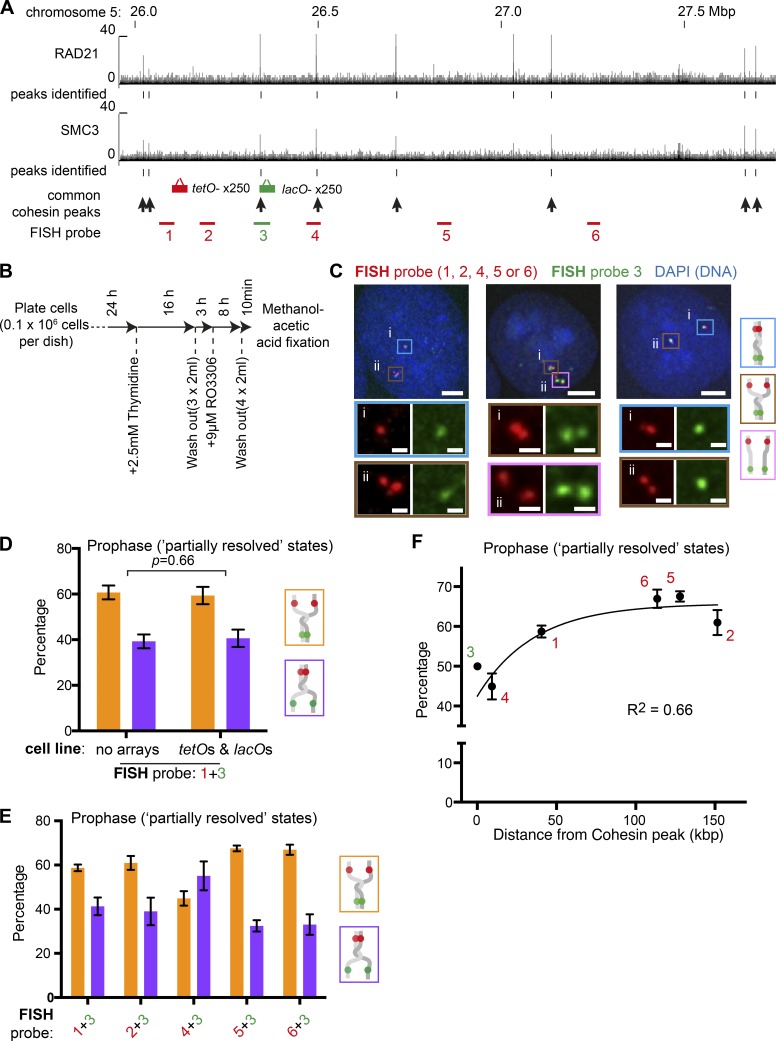
**FISH results suggest correlation between local cohesin enrichment and robust sister chromatid cohesion during prophase. (A)** The ChIP-seq data (ENCODE Project Consortium, 2012) show the distribution of RAD21 and SMC3 along the genomic region around insertion sites of *lacO* and *tetO*. Common enrichment sites of RAD21 and SMC3 are indicated by black arrows. The regions against which FISH probes were generated are indicated as red or green bars. **(B)** Experimental procedure outline. Cells were arrested in S phase using thymidine, released, and subsequently arrested at the G2-M phase boundary using the CDK1 inhibitor RO-3306. Prophase cells were fixed 10 min after washout of RO-3306. **(C)** Fixed prophase cells were hybridized with the indicated FISH probes (their positions are shown in A). Zoomed regions for each cluster of hybridized probes, corresponding to i or ii in the panel above, are shown below with the frames color-coded according to the key on the right. Scale bars, 5 µm (top) and 1 µm (bottom; zoomed images). **(D)** Graph shows the proportion of sister separation between FISH probes 1 (orange bars) and 3 (purple bars) shown in A among all “partial resolution” states for different prophase cells either containing no operator array (WT HT-1080) or containing *tetO* and *lacO* (TT75). Mean and standard error are shown from three independent experiments (*n* = 150–249 FISH signal clusters were analyzed in each experiment). P values were obtained by chi-square test. **(E)** Graph shows the proportion of sister separation between indicated pairs of FISH probes (shown in A) among all “partial resolution” states in prophase of WT HT-1080 cells (no *tetO* or *lacO*). Mean and standard error are shown from four to seven independent experiments for each FISH probe pair (*n* = 134–416 FISH signal clusters were analyzed in each experiment). **(F)** Sister chromatid separation rates for each FISH probe, plotted against distance to the center of the nearest cohesin peak indicated in A. Mean and standard error percentage sister separation was plotted for FISH probes 1, 2, 4, 5, and 6 (as in E). These probes were used with probe 3 in E. To represent separation of probe 3 for comparison with the other probes, we plotted a fixed value of 50%. This is the theoretical rate of separation for any probe when mixed with an identical probe for this experiment. The data points were fit to an exponential one-phase decay curve, and the R^2^ value shows closeness of the fit.

Next, to study sister chromatid resolution and separation at more chromosome sites, we used FISH. Using FISH probes set in the region including *tetO* and *lacO* integration sites (Fig. 5 A), we investigated sister chromatid separation in HT-1080 cells fixed at prophase (Fig. 5 B). To exclude possible off-target signals in FISH, we used two probes (with different colors) together and analyzed their signals only if they locate in close proximity (Fig. 5 C). Focusing on “partially resolved” states (i.e., one of the two probes showing separation), we first compared the frequency of sister separation of FISH probes in cell lines with or without *tetO* and *lacO* integration. Both conditions gave very similar results, in which Probe 1 (close to *tetO* integration site) showed higher sister separation than probe 3 (at *lacO* integration sites; Fig. 5, A and D). The *lacO* integration site (probe 3) showed higher percentage separation with FISH than with live-cell imaging (Fig. 4 A), which is probably due to (a) harsher treatment of cells during FISH preparation and/or (b) Probe 1 being closer (than the *tetO* integration site) to small cohesin peaks (∼26.05 Mbp on chromosome 5; Fig. 5 A), leading to a relatively low percentage separation of probe 1 (thus, a relatively high percentage separation of probe 3). We conclude that integration of *tetO* and *lacO* did not affect sister separation frequency of their integration sites. We then compared sister separation frequency using various FISH probes in prophase cells without *tetO* and *lacO* arrays in “partially resolved” states (Fig. 5 E). As shown in Fig. 5 F, larger distance from cohesin peaks (marked by arrows in Fig. 5 A) correlated well with higher sister separation frequency. This suggests that local cohesin peaks delay sister chromatid separation around these peaks during prophase.

### Sister chromatid resolution is established in prophase and maintained during prometaphase, relying on topo II activity

**Figure 6.**
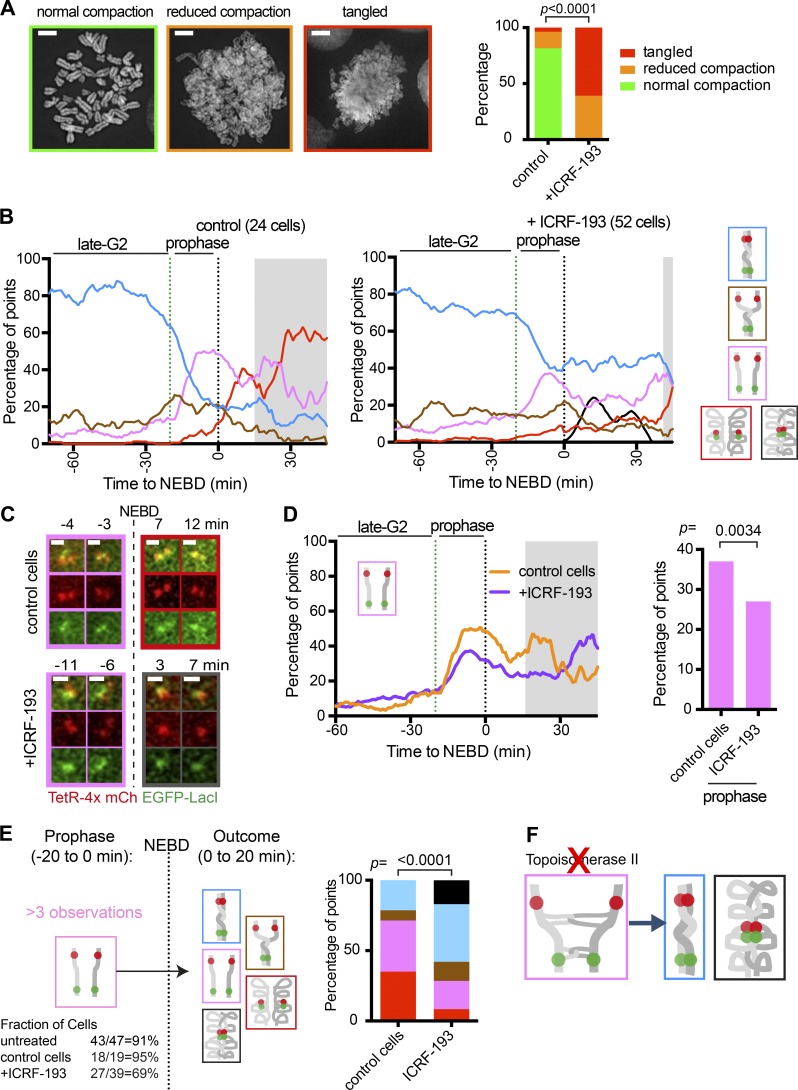
**Topo II is required to stabilize resolved sister chromatids during prometaphase. (A)** Spread of metaphase chromosomes from ICRF-193–treated and control cells. Representative metaphase spreads are shown on the left with the frame of the color coding. Scale bars, 5 µm. The proportion of each class of spread is shown on the right (*n* = 27 for control and 23 for ICRF-193 treatment). The P value was obtained by the chi-square test. **(B)** The proportion of each configuration of the fluorescence reporter in control and ICRF-193–treated cells. TT75 cells were arrested by a double-thymidine block, released, treated with MK1775 and ICRF-193 (or only with MK1775 for control), and observed by live-cell microscopy, as in Fig. S4 A. Data from individual cells are shown in Fig. S4 B. The number of analyzed cells at each time point was between 10 and 21 (mean: 16) for control; 10 and 46 (mean: 30) for ICRF-193, except for the gray-shaded areas where <10 cells were analyzed. **(C)** Representative live-cell images of the fluorescence reporter in a control or ICRF-193–treated cell. The frame colors match the color coding in B. **(D)** The proportion of the pink “resolved” state for control or ICRF-193–treated cells plotted against time (left) or during prophase (right). These data were taken from B. The gray-shaded area is as in Fig. 3 F. P value was obtained by chi-square test (*n* = 366 and 961 for control and ICRF-193–treated cells, respectively). **(E)** The change in the fluorescence reporter configuration following NEBD. Diagram (left) shows the pipeline of assessment; cells with the pink “resolved” state for four or more time points during prophase (the fraction of such cells shown at bottom) were assessed further. In such cells, configurations during the 20 min following NEBD were scored in control (*n* = 142 time points) and ICRF-193–treated (*n* = 254) cells (right). P value was obtained by chi-square test. **(F)** Diagram explaining the outcome with the inhibited topo II activity.

Using our live-cell assay system, we next studied how chromosome reorganization in early mitosis is affected by the specific catalytic inhibitor of topo II, ICRF-193 (Ishida et al., 1991). We first confirmed that, following ICRF-193 treatment, chromosomes looked tangled in the majority of metaphase cells (Fig. 6 A); thus, sister chromatid resolution was indeed defective, after topo II inhibition, as previously reported (Ishida et al., 1991).

We then compared the change in configuration of fluorescent dots from late G2 to early mitosis in the presence and absence of ICRF-193 (Fig. 6, B and C; and Fig. S4, A–C). Since ICRF-193 treatment leads to engagement of the G2/M checkpoint (Downes et al., 1994; Ishida et al., 1994), we bypassed the checkpoint by using the WEE1 inhibitor MK-1775 (Hirai et al., 2009; Fig. S4 A). MK-1775 treatment itself (control) did not significantly affect the change in configuration of fluorescent dots (Fig. 6 B, left; compare with Fig. 2 D). In contrast, treatment with both ICRF-193 and MK-1775 (simply designated ICRF-193 treatment below) caused mild reduction in the pink “resolved” state during prophase (Fig. 6 D). ICRF-193 treatment also led to colocalization of all four fluorescent dots after NEBD (black in Fig. 6 B, right), which we interpret as an abnormal “nonresolved and compacted” state (Fig. S4 C). Moreover, along the time course of individual ICRF-193–treated cells, the pink “resolved” state often reverted to the blue (and black) “nonresolved” state after NEBD (Fig. 6, C [bottom] and E). When topo II activity is reduced, DNA catenation may still remain after overall completion of sister chromatid resolution in this region, and may subsequently destabilize largely resolved sisters, leading to such reversion (Fig. 6 F).

This ICRF-193 phenotype was also reproduced without using the WEE1 inhibitor: that is, by releasing cells from G2/M arrest (which bypassed G2/M checkpoint) and adding ICRF-193 (Fig. S4, E–G). Overall, topo II activity is required to resolve sister chromatids in prophase, as observed by others (Giménez-Abián et al., 1995; Liang et al., 2015; Nagasaka et al., 2016). In addition, we have found a novel role of topo II in stabilizing and maintaining resolved sister chromatids during prometaphase. This finding is consistent with the recent report that topo II–dependent sister chromatid resolution could be reversible (Piskadlo et al., 2017).

### Distinct roles of condensin I and II in sister chromatid resolution and chromosome compaction

**Figure 7.**
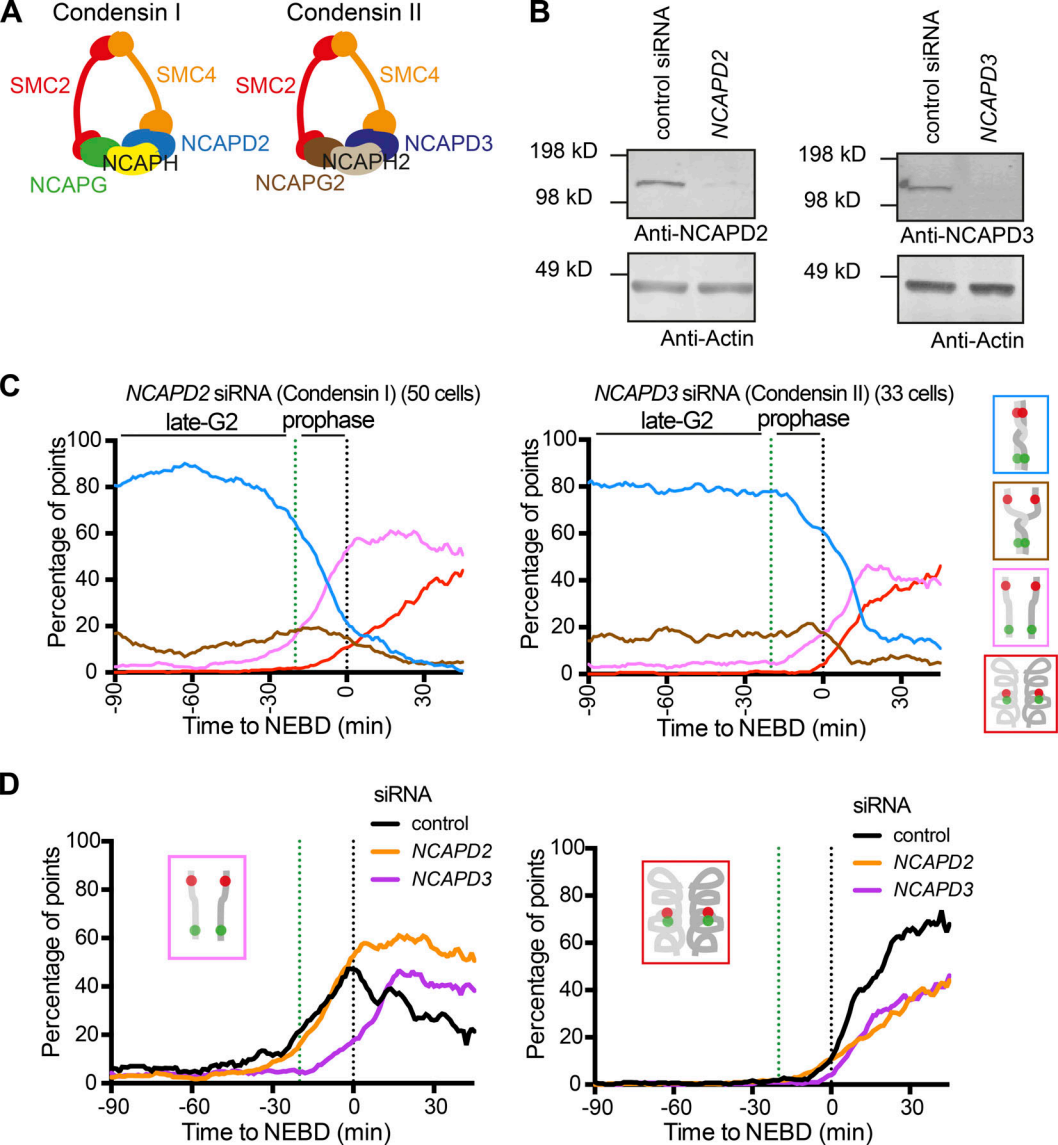
**Condensin I and II play distinct roles in sister chromatid resolution and compaction. (A)** Diagram showing composition of condensin I and II. They contain common subunits SMC2 and SMC4 and unique subunits NCAPG/H/D2 (condensin I) or NCAPG2/H2/D3 (condensin II). **(B)** Western blotting of NCAPD2 (condensin I; left) or NCAPD3 (condensin II; right) proteins following treatment with the indicated siRNA for 48 h. Actin is shown as a loading control. **(C)** The proportion of each configuration of the fluorescence reporter in cells depleted of NCAPD2 or NCAPD3. TT75 cells were treated and analyzed as in Fig. S2 I. Data from individual cells are shown in Fig. S5 A. The number of analyzed cells at each point is between 10 and 44 (mean: 26) for NCAPD2 siRNA; 10 and 36 (mean: 23) for NCAPD3 siRNA. **(D)** The proportion of the pink “resolved” (left) or red “compacted” (right) state is compared over time between control, NCAPD2, and NCAPD3 siRNA. These data were taken from Figs. 3 E and 7 C.

Next, we investigated the roles of condensin I and II with our assay. To deplete cells of either condensin I or II, we used siRNAs against NCAPD2 or NCAPD3, respectively (Fig. 7 A). Their depletion was confirmed by Western blotting (Fig. 7 B). We characterized the configuration of the fluorescent dots over time (Fig. S5, A and B) and plotted proportions of each configuration (Fig. 7 C; compare with control siRNA in Fig. 3 E). NCAPD2-depleted cells showed appearance of the pink “resolved” state during prophase, with a similar timing to cells treated with a control siRNA (Fig. 7 D, left). In contrast, NCAPD3-depleted cells showed a delay in the appearance of the pink state compared with control cells (Fig. 7 D, left). Intriguingly, both NCAPD2- and NCAPD3-depleted cells showed a delay in the appearance of the red “compacted” state, relative to control cells; the extent of this delay was similar in NCAPD2- and NCAPD3-depleted cells (Fig. 7 D, right).

Thus, NCAPD3 (condensin II)-depleted cells show a defect in sister chromatid resolution. In contrast, NCAPD2 (condensin I)-depleted cells showed no delay in sister chromatid resolution but did show a delay in chromosome compaction. Therefore, condensin II and I play distinct roles in sister chromatid resolution and chromosome compaction, which is consistent with previous reports (Hirano, 2012; Nagasaka et al., 2016). However, to address the exact extent of condensin II and I in promoting sister chromatid resolution and chromosome compaction, or their relative contribution in facilitating these processes, we need to understand the kinetics of these processes more quantitatively.

### Quantitative analyses reveal the specific timing and progression of chromatid resolution and chromosome compaction promoted by condensin II and I, respectively

**Figure 8.**
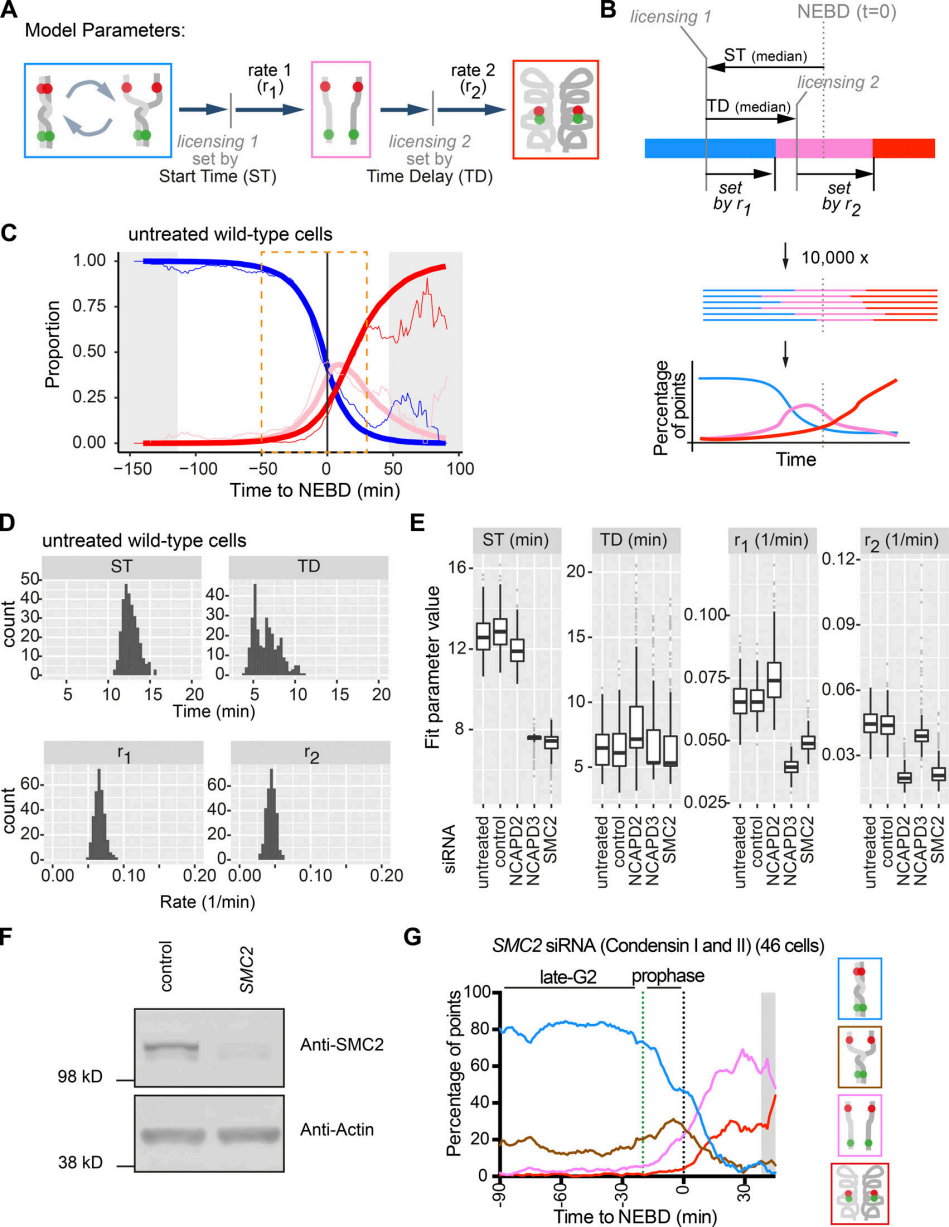
**Mathematical modeling of sister chromosome resolution and compaction. (A)** Diagram illustrates setting of four parameters: ST, TD, r_1_, and r_2_. Also refer to B (top) for how licensing 1 and 2 were defined by ST and TD. **(B)** Schematic shows the procedure of modeling. Top: shows how the timing of transitions was defined by four parameters (see A). The color codes represent the chromosome configurations as in A. Note that, in a subset of cells, the transition to the pink “resolved” state happened after licensing 2; in these cells, transition to the red “compacted” state could only occur after the transition to the pink state. **(C)** Microscopy data (thin curves; from Fig. 2 C) and the best-fit model (thick curves) for untreated WT cells. The curves represent the proportion of each configuration of the fluorescence reporter over time. The microscopy data curves were smoothed over 5 min, while model fitting was performed on original nonsmoothed data (within the orange dotted box). The color codes are as in A and gray-shaded area is as in Fig. 3 E. **(D)** Distributions of best-fit parameter values were obtained from 300 bootstrapping repetitions for untreated WT cells. **(E)** Box-and-whisker plots of best-fit parameter values, obtained from bootstrapping (see D), for the indicated siRNA conditions. Thick horizontal lines show medians. **(F)** Western blotting analysis of SMC2 protein following treatment with SMC2 or control siRNA for 48 h. Actin is shown as a loading control. **(G)** The proportion of each configuration of the fluorescence reporter in cells depleted of SMC2. TT75 cells were treated and analyzed as in Fig. S2 I. Data from individual cells are shown in Fig. S5 A. The number of analyzed cells at each time point is between 10 and 56 (mean: 38), except for the gray-shaded areas where <10 cells were analyzed.

To analyze kinetics of sister chromatid resolution and chromosome compaction more quantitatively, we developed a stochastic model that describes the following two transitions in configuration of fluorescent dots: (a) from the blue “nonresolved” state (including the brown “partially resolved” state) to the pink “resolved” state and (b) from the pink to the red “compacted” state (Fig. 8 A). Each of the two transitions followed a similar sequence of events. First, cells became licensed for change with timing defined by the start time (ST) for transition (a) or the time delay (TD) for transition (b). In each case, after licensing, the relevant transition occurred stochastically with the constant rate r_1_ for transition (a) or r_2_ for transition (b). ST and TD defined median time of each licensing event, as shown in Fig. 8 B (top). A larger ST value meant that sister chromatid resolution started earlier, while a larger TD value meant that chromosome compaction began with a larger delay after sister chromatid resolution (Fig. 8 B). For a given set of parameter values, the simulation generated a sequence of states over time, and from 10,000 simulations, proportions of blue, pink, and red states were obtained (Fig. 8 B). The best-fitting model parameter values were found by minimizing the mean-square difference between the simulation and the microscopy data (calculated between −50 and +30 min, relative to NEBD; Fig. 8 C). Uncertainties of the best-fitting parameter values were estimated by bootstrapping microscopy data, with the median reported as the central estimate of the parameter value (Fig. 8, D and E).

As expected, the best-fitting parameter values obtained for untreated and control siRNA conditions were very similar (Fig. 8 E). In these WT cells, values of ST and TD were 12–13 min and 6–7 min, respectively, suggesting that the final stage of sister chromatid resolution (transition to the pink “resolved” state) starts 12–13 min before NEBD and chromosome compaction (transition to the red “compacted” state) begins 6–7 min later. In NCAPD3-depleted cells, ST was reduced by 5–6 min and r_1_ was reduced by ∼40% relative to the control, which suggests a delay and inefficiency in sister chromatid resolution (Fig. 8 E and Fig. S5, C and D). Nonetheless, in NCAPD3-depleted cells, TD was almost normal and, once chromosome compaction started, it proceeded with almost normal kinetics since r_2_ was almost unchanged.

In contrast, NCAPD2-depleted cells showed similar values of ST and r_1_ to control values, indicating largely normal sister chromatid resolution (Fig. 8 E and Fig. S5, C and D). In NCAPD2-depleted cells, TD was also similar to control values but r_2_ was reduced by ∼55%, suggesting no significant delay but considerable inefficiency in chromosome compaction. Thus, condensin II (NCAPD3) and condensin I (NCAPD2) specifically promote sister chromatid resolution and chromosome compaction, respectively, and our modeling quantified the extent of defects in these processes when each factor was depleted.

To validate the above mathematical model, we made the following prediction: if we deplete SMC2, which is a common subunit of condensin I and II (Fig. 7 A), we would see the combined phenotypes of NCAPD2- and NCAPD3-depleted cells (i.e., the combined changes in parameter values). To test this prediction, we depleted SMC2 using siRNA, which was confirmed by Western blotting (Fig. 8 F). We then analyzed the configuration of fluorescent dots by microscopy in the SMC2-depleted cells (Fig. 8 G and Fig. S5, A–C), applied the above mathematical model, and fitted parameter values for ST, TD, r_1_, and r_2_ (Fig. 8 E and Fig. S5 D). In SMC2-depleted cells, the values of ST and r_1_ were similar to those in NCAPD3-depleted cells while the value of r_2_ was similar to that in NCAPD2-depleted cells—in other words, when either NCAPD2- or NCAPD3-depleted cells showed changes in parameter values (from those in controls), SMC2-depleted cells also showed very similar changes in these parameter values (Fig. 8 E). Thus, SMC2-depleted cells showed the combined defects of NCAPD2- and NCAPD3-depleted cells, when their defects were described by changes in parameter values by modeling. Therefore, we can effectively predict the defects of SMC2 depletion from the individual defects of NCAPD2 and NCAPD3 depletions. The results suggest that our mathematical model indeed provides accurate parameter values to represent timing and efficiency of sister chromatid resolution and chromosome compaction.

## Discussion

To analyze dynamics of sister chromatid resolution and chromosome compaction, we developed a novel real-time assay using fluorescence reporters. Compared with other assays, ours has the following three major advantages: First, our assay allows analyses of chromosome configuration with higher spatial resolution compared with bulk chromosome assays. We visualized two neighboring chromosome sites (100–250-kbp interval) as small fluorescent dots and investigated the change in their 3D configuration over time. Observation with high spatial resolution allows analyses of dynamic local chromosome changes. Second, our assay allows analyses with high temporal resolution; we acquired live-cell images every minute and aligned time-course data of individual cells relative to NEBD. By observing events in individual cells with high temporal resolution, we can analyze and quantify rapidly changing events. Third, our method allows analyses of both sister chromatid resolution and chromosome compaction in one assay. By analyzing both together, we can address their relative timing and potential coordination. Other assays usually focus on only one of the two; for example, Hi-C analyses enable detailed genome-wide study of chromosome compaction but provide little information about sister chromatid resolution. Taking these advantages of our assay, we found novel regulation and dynamics of mitotic chromosome organization as highlighted below.

First, we found dynamics of sister chromatid resolution in late G2 phase and prophase. Sister chromatid resolution begins in late G2 phase (Ono et al., 2013; Stanyte et al., 2018) and continues into prophase (Nagasaka et al., 2016). Stanyte et al. (2018) reported that sister chromatids repetitively cycle through unresolved and partially resolved states in G2 with ∼30-min intervals. We showed that this repetitive cycle occurs even more dynamically (i.e., with an interval of a few minutes). The cyclical behavior we observe might reflect multiple attempts to remove the stably bound cohesin from chromosomes. It could also reflect the motion of a dynamic population of cohesin moving locally along chromosomes (Ocampo-Hafalla et al., 2016; Busslinger et al., 2017). It is also possible that cohesin-dependent chromatin loops are removed in late G2, which could enhance dynamic sister chromatid resolution (Nozaki et al., 2017; Rao et al., 2017).

Second, we found that local accumulation of cohesins along chromosome arms correlates with the robustness of sister chromatid cohesion. Moreover, a local reduction in cohesin accumulation by deletion of CTCF binding sites led to precocious sister chromatid resolution in prophase. As far as we know, these results provide the first evidence that enriched cohesins at a chromosome site in noncentromeric regions locally contribute to robust cohesion. Recently, Stanyte et al. (2018) reported that the extent of sister chromatid separation in G2 phase is not correlated with the distance from cohesin accumulation sites on several chromosomes but is rather correlated with other properties of chromosome domains such as replication timing. We may have been able to detect correlation between cohesin accumulation and robustness of cohesion since we focused on one chromosome region where other properties are supposedly common. Meanwhile, although it was known that cohesins enriched at CTCF binding sites promote intrachromatid looping during interphase (Dixon et al., 2012; Rao et al., 2014), it was unknown if they also support sister chromatid cohesion. Our results provide the first evidence for this notion. Cohesins at CTCF binding sites may switch their roles between intrachromatin looping and sister chromatid cohesion. It will be intriguing to study whether such switching indeed occurs and, if so, how it is regulated.

Third, by analyzing both sister chromatid resolution and chromosome compaction in one assay, we successfully evaluated relative timing and regulation of the two events. Moreover, by fitting computer models to live-cell imaging data, we were able to quantify timing and progression of the two events. On average, resolution begins 12–13 min before NEBD and compaction starts 6–7 min later. Condensin II and condensin I promote sister chromatid resolution and chromosome compaction, respectively. Our modeling allowed us to quantify contribution of condensin II and I to each process. Recent computer modeling suggests that chromatin looping could drive not only chromosome compaction but also sister chromatid resolution (Goloborodko et al., 2016). If so, defects in the resolution with condensin II depletion, but not with condensin I depletion (Fig. 8 E), could be explained by the observation that condensin II promotes chromatin looping earlier than does condensin I (Gibcus et al., 2018). Meanwhile, defects in the compaction with condensin I depletion, but not with condensin II depletion (Fig. 8 E), could be explained by the result that condensin II and I promote large and small chromatin looping, leading to chromosome axial shortening and chromosome compaction, respectively (Gibcus et al., 2018). Overall, our results provide an important framework for temporal and molecular regulation of sister chromatid resolution and chromosome compaction.

To visualize selected chromosome loci, we used *tetO* and *lacO* inserted at targeted chromosome loci. Alternative methods for labeling selected chromosome loci in live cells use the nuclease-deficient Cas9 (dCas9) and single guide RNAs (sgRNAs; Chen et al., 2013; Ma et al., 2015). By transient transfection of different sgRNAs, the dCas9/sgRNA method would allow us to rapidly analyze more chromosome regions. However, this method has been mainly used to visualize repetitive DNA sequences that provide workable signal intensities (Stanyte et al., 2018). Since our assay relies on visualization of two neighboring chromosomal loci (100–250 kb apart), and suitably spaced unique repetitive sequences are not available, dCas9/sgRNA methods are not currently suitable to carry out the experiments we described. Nonetheless, the sensitivity of dCas9/sgRNA methods is improving rapidly (Maass et al., 2018; Neguembor et al., 2018) and may allow more routine visualization of nonrepetitive sequences in the near future. Analyses of a larger number of chromosome regions in various cells and in various conditions with live-cell imaging would broaden our knowledge about mitotic chromosome organization.

## Materials and methods

### Cell culture

The human cell line HT-1080 (obtained from American Type Culture Collection) and derivative cell lines were cultured at 37°C and 5.0% CO_2_ under humidified conditions in DMEM (with l-glutamine), 10% FBS, 100 U/ml penicillin, and 100 µg/ml streptomycin. Media and supplements were obtained from Invitrogen. For microscopy, the above medium was replaced with Fluorobrite DMEM medium (Invitrogen) supplemented with 10% FBS, 2 mM l-glutamine, 1 mM pyruvate, and 25 mM Hepes.

The DT40 cell line BM-lacO-20K-19 (Hori et al., 2013) and derivative cell lines were cultured at 37°C and 5.0% CO_2_ under humidified conditions in RPMI-1640 (with l-glutamine), 10% FBS, 1% chicken serum, 100 U/ml penicillin, and 100 µg/ml streptomycin. Media and supplements were obtained from either Invitrogen or Lonza. For microscopy, the above medium was replaced with colorless RPMI-1640 (phenol red–free) medium supplemented with 10% FBS, 1% chicken serum, 2 mM l-glutamine, 1 mM pyruvate, and 25 mM Hepes.

Transfection of plasmids into HT-1080 and derivative cell lines was facilitated with Fugene HD according to the manufacturer’s instructions (Promega). Cells were transfected in single wells of a 6-well dish using 4.5 µl Fugene HD and 1.5 µg plasmid (3:1 ratio). Selection was performed 24–48 h after transfection using puromycin (Sigma; 0.3 µg/ml), blasticidin (Invivogen; 2 µg/ml), hygromycin (Roche; 60 µg/ml), histidinol (Sigma; 2 mg/ml), and G418 (Sigma; 300 µg/ml).

Transfection of DT40 cells was performed by electroporation of 1.0 × 10^7^ cells with 10–15 mg linearized plasmid using 0.4 cm Gene Pulsar cuvettes and Gene Pulsar electroporation apparatus (Bio-Rad) at 550 V and 25 mF. Selection was performed using puromycin (final concentration: 0.5 µg/ml) or histidinol (final concentration: 1 mg/ml).

Protein knockdown by siRNA was performed using lipofectamine (Invitrogen) according to the manufacturer’s instructions. In all cases, 0.01 nmol siRNA with 6 µl lipofectamine and 200 µl Optimem (Invitrogen) were added to cells in 2 ml of medium in 6-well or 3-cm microscopy dishes. The medium containing the siRNA was replaced and fresh siRNA added every 24 h. Cells for analysis by Western blotting or microscopy were used between 48 and 60 h after the first addition of siRNA. siRNAs were obtained from Ambion or Eurofins. The sequences were as follows; WAPL (5′-CGGACUACCCUUAGCACAAdTdT-3′); RAD21 (5′-AUACCUUCUUGCAGACUGUdTdT-3′); NCAPD2 (5′-CGU​AAG​AUG​CUU​GAC​AAU​UTT-3′); NCAPD3 (no. 1, 5′-GAU​AAA​UCA​GAG​UAU​CGU​ATT-3′, or no. 2, 5′-GAA​CAG​CGA​UUC​AAC​AUC​ATT-3′); SMC2 (no. 1, 5′-GAAUUAGACCACAACAUCAdTdT-3′, or no. 2, 5′-CUAUCACUCUGGACCUGGAdTdT-3′); control (nonspecific; 5′-UAA​CGA​CGC​GAC​GAC​GUA​ATT-3′).

Synchronization of human cells at the G1/S phase boundary was achieved using a double-thymidine block (Fig. S2 I). Essentially, 0.10 × 10^6^ cells were seeded in 2 ml of medium in 6-well dishes or 3-cm glass-bottomed microscopy dishes (World Precision Instruments) 16 to 24 h before the treatment. Thymidine was then added at a final concentration of 2.5 mM and incubated for 16 h. Thymidine was then removed and cells were washed 3 × 2 ml with fresh medium. Cells were incubated for a further 8 h before 2.5 mM thymidine was added. Cells were then incubated for 12–16 h and thymidine was then removed and cells were washed 3 × 2 ml with fresh medium and incubated for a further 5–10 h to observe cells in late S phase or G2/M phase, as appropriate.

Synchronization of human cells at the G2/M phase boundary was achieved using the CDK1-inhibitor RO-3306 (Millipore; Fig. S4 E). Essentially, 0.10 × 10^6^ cells were seeded in 2 ml of medium in 6-well dishes or 3-cm glass-bottomed microscopy dishes (World Precision Instruments) 16–24 h before the treatment. RO-3306 was then added at a final concentration of 9 µM and cells were incubated for 12 h. RO-3306 was then removed and cells were washed with 4 × 2 ml PBS before 2 ml fresh medium was added.

The topo II inhibitor ICRF-193 (Sigma) was used at a concentration of 2 µg/ml and the WEE1 inhibitor MK1775 (Selleckchem) was used at a concentration of 0.5 µM. The DNA stain SiR-DNA (Tebu-bio) was used at a concentration of 200 nM and was added to cells ≤12 h before imaging.

### Plasmids

For integrating *tet* operator arrays into human chromosome 5, the plasmids pT2770 and pT2707 were used. pT2707 contains the Cas9 gene and the specific guide DNA (sgDNA; sequence: 5′-ACG​GGT​TCT​TGT​CCG​TCC​CA-3′), which was cloned into pGeneArt-CRISPR-nuclease according to the manufacturer’s instructions (Invitrogen; A1175). pT2770 was created to target the *tet* operator arrays to the region and contained homology to genomic regions upstream and downstream of the selected gDNA site of pT2707. The homology arms were amplified from genomic DNA by PCR using primer pairs Chr5-26Mb-5′F (5′-AAA​AAA​CTC​GAG​CGA​TCG​TGT​CTT​GGG​GAT​GTT​CCA​CGG​GTA​C-3′) and Chr5-26Mb-5′R (5′-AAA​AAA​AGA​TCT​TTG​GAT​CCA​CGG​ACA​AGA​ACC​CGT​CTT​CAG​CTG-3′), and Chr5-26Mb-3′F (5′-AAA​AAA​GGA​TCC​TTA​GAT​CTC​CCA​CGG​CCA​TGA​AAA​TGT​GGG​CTC-3′) and Chr5-26Mb-3′R (5′-AAA​AAA​GCG​GCC​GCC​TTT​CTT​GAC​ACA​TTG​TTG​GGA​ACC-3′) and sequentially cloned into ploxPuro (Arakawa et al., 2001). *tet* operator arrays (250 repeats; 9.1 kb) with nonrepetitive 10-bp DNA sequences between each repeat from pLAU44 (Lau et al., 2003) and the puromycin resistance gene (from ploxPuro) were then sequentially cloned in between the regions of homology to create pT2770. At each stage, restriction digestion and sequencing were used to confirm cloning.

For integrating *lac* operator arrays into human chromosome 5, 250 kb upstream of the *tet* operator arrays, the plasmids pT2846 and pT2837 were used. pT2837 contains the Cas9 gene and the sgDNA (sequence: 5′-CAT​TTA​GGT​TTT​TCA​CGT​AC-3′) was cloned into pGeneArt-CRISPR-nuclease according to the manufacturer’s instructions (Invitrogen; A1175). pT2846 was created to target the *lac* operator arrays to the region and contained homology to genomic regions upstream and downstream of the selected gDNA site of pT2837. The homology arms were amplified from genomic DNA by PCR using primer pairs 5Chr5-26Mbii-F (5′-AAA​AAC​TCG​AGA​TGC​TAA​GTG​TGG​GAG​GGC​AAT​TTC-3′) and 5Chr5-26Mbii-R (5′-AAA​AAG​GAT​CCT​TGT​CGA​CGT​ACT​GGG​ATA​ATA​GGA​ACA​TTT​GAA​AC-3′), and 3Chr5-26Mbii-F (5′-AAA​AAA​GGA​TCC​TTA​GAT​CTG​TGA​AAA​ACC​TAA​ATG​ACA​CCA​TCA​CC-3′) and 3Chr5-26Mbii-R (5′-AAA​AAA​GCG​GCC​GCC​TGC​CTC​TCT​CTC​TCA​TAC​ACA​TGT​G-3′) and sequentially cloned into ploxBlast (Arakawa et al., 2001). *lac* operator arrays (250 repeats; 9.6 kb) with nonrepetitive 10-bp DNA sequences between each repeat from pLAU43 (Lau et al., 2003) and the blasticidin resistance gene (from ploxBlast) were then sequentially cloned in between the regions of homology to create pT2846. At each stage, restriction digestion and sequencing were used to confirm cloning.

For integrating *lac* operator arrays into human chromosome 5, 750 kb upstream of the *tet* operator arrays, the plasmids pT2838 and pT2847 were used. pT2838 contains the Cas9 gene and the sgDNA (sequence: 5′-TAG​GCT​TCA​CCG​TAG​TAT​CT-3′) was cloned into pGeneArt-CRISPR-nuclease according to the manufacturer’s instructions (Invitrogen; A1175). pT2847 was created to target the *lac* operator arrays to the region and contained homology to genomic regions upstream and downstream of the selected gDNA site of pT2838. The homology arms were amplified from genomic DNA by PCR using primer pairs 5Chr5-26Mb-Fiii (5′-AAA​AAC​TCG​AGA​TTA​ACT​TCC​ACT​ACT​CTA​CTA​GAG​CTG-3′) and 5Chr5-26Mb-Riii (5′-AAA​AAG​GAT​CCT​TGT​CGA​CAT​CTT​GGA​TAC​TAC​CTA​CGT​ATG​TAT​G-3′), and 3Chr5-26Mb-Fiii (5′-AAA​AAA​GGA​TCC​TTA​GAT​CTA​CTA​CGG​TGA​AGC​CTA​CAT​AGA​C-3′) and 3Chr5-26Mb-Riii (5′-AAA​AAA​GCG​GCC​GCC​ACT​GTA​TTA​TTT​TCC​TAG​AGC​TGC​CC-3′) and sequentially cloned into ploxBlast (Arakawa et al., 2001). *lac* operator arrays (250 repeats; 9.6 kb) with nonrepetitive 10-bp DNA sequences between each repeat from pLAU43 (Lau et al., 2003) and the blasticidin resistance gene (from ploxBlast) were then sequentially cloned in between the regions of homology to create pT2847. At each stage, restriction digestion and sequencing were used to confirm cloning.

For integrating *tet* operator arrays into the chicken (DT40) Z chromosome, the plasmid pHH100TetO was used. pHH100TetO contains homology to genomic regions upstream and downstream of the selected target site. The homology arms were amplified from genomic DNA by PCR using primer pairs 100TetLA5Not (5′-TAT​AGC​GGC​CGC​CCT​CAG​ATT​GTT​CAA​ACA​TTA​ATG​AGA​TGC-3′) and 100TetLA3Bgl (5′-ATA​AGA​TCT​GGA​TCC​CCA​TAT​CTG​AAA​TCC​AAA​TGT​TTA​CAA​AAT-3′), and 100TetRA5Bgl (5′-TAT​AAG​ATC​TAC​AAC​CTA​TTG​AGC​AGT​TGA​AGG​TGG​AAG​G-3′) and 100TetRA3Xho (5′-TAT​ACT​CGA​GGC​TAG​TGC​TGC​TGG​ATT​ATC​CAG​AAG​CTC​C-3′) and sequentially cloned into pBLUESCRIPT. Next, *tet* operator arrays (250 repeats; 9.1 kb) from pLAU44 (Lau et al., 2003) and the puromycin resistance gene were sequentially cloned in between the regions of homology to create pHH100TetO.

For visualizing *tet* operator arrays in live human or chicken cells, a plasmid expressing TetR-4mCherry under the control of the β-actin promoter was created. For this, *tetR* (Michaelis et al., 1997) and four copies of mCherry (Renshaw et al., 2010) were cloned into pExpress (Arakawa et al., 2001) along with the histidinol resistance gene from pJE59 (Eykelenboom et al., 2013). Restriction digestion and sequencing were used to confirm cloning.

For visualizing *lac* operator arrays in live human or chicken cells, the plasmid pEGFP-lacI-NLS was used (Hori et al., 2013). For visualizing replication factories in live human cells, the plasmid pmCerulean-PCNA-19-SV40NLS-4 was used and was a gift from the Davidson laboratory (Addgene plasmid 55386).

For deleting the specific CTCF-binding site in human cells (detailed in the Results and Fig. 4 C), the plasmids pT3093 and p3099 were used. pT3093 and pT3099 contain the Cas9 gene and the sgDNAs (sequences: 5′-GAC​TTA​GTC​CCT​ACC​TCA​CA-3′ and 5′-AAT​CAC​TGT​GAG​CCT​GCC​TA-3′, respectively), which were cloned separately into pGeneArt-CRISPR-nuclease according to the manufacturer’s instructions (Invitrogen; A1175). pT3093 and pT3099 were designed to cleave upstream and downstream of the targeted CTCF-binding site, respectively.

### Cell lines

The human HT-1080–derived cell line containing *tet* operator and *lac* operator arrays, separated by 250 kbp of DNA and expressing TetR-4mCherry and EGFP-LacI was designated TT75 and was created as follows. HT-1080 cells were transfected with pT2707 and pT2770 and puromycin-resistant clones were obtained. Targeting of the *tet* operator arrays to the desired genomic location was confirmed using the primers 5F (5′-CTT​GTG​ACA​TGA​CCT​TCT​AAA​TAG​AGT​GC-3′), 5R (5′-CAC​TGC​ATT​CTA​GTT​GTG​GTT​TGT​CC-3′), 5ctrlF (5′-AAA​AAA​CTC​GAG​CGA​TCG​TGT​CTT​GGG​GAT​GTT​CCA​CGG​GTA​C-3′), 5ctrlR (5′-AAA​AAA​AGA​TCT​TTG​GAT​CCA​CGG​ACA​AGA​ACC​CGT​CTT​CAG​CTG-3′), 3F (5′-GCC​CTG​ATC​AAT​AAC​TTC​GTA​TAA​TG), 3R (5′-CTC​AAC​AGA​AGA​CCT​CCT​GTT​GCT​C-3′), 3ctrlF (5′-AAA​AAA​GGA​TCC​TTA​GAT​CTC​CCA​CGG​CCA​TGA​AAA​TGT​GGG​CTC-3′), and 3ctrlR (5′-AAA​AAA​GCG​GCC​GCC​TTT​CTT​GAC​ACA​TTG​TTG​GGA​ACC-3′). This cell line was designated TT51. This cell line was then sequentially transfected with pT2415 and pEGFP-LacI-NLS with selection for histidinol and hygromycin resistance, respectively. Finally, these cells were transfected with pT2837 and pT2846 and blasticidin-resistant clones were obtained. Targeting of the *lac* operator arrays to the desired genomic location was confirmed using the primers 5Fii (5′-GCA​GGT​GCA​TGG​GAA​TAC​AAG​TGT​TG-3′), 5Rii (5′-CTC​ATC​AAT​GTA​TCT​TAT​CAT​GTC​TGG​ATC-3′), 5ctrlFii (5′-AAA​AAC​TCG​AGA​TGC​TAA​GTG​TGG​GAG​GGC​AAT​TTC-3′), and 5ctrlRii (5′-AAA​AAG​GAT​CCT​TGT​CGA​CGT​ACT​GGG​ATA​ATA​GGA​ACA​TTT​GAA​AC-3′), 3Fii (5′-GCC​CTG​ATC​AAT​AAC​TTC​GTA​TAA​TG), 3Rii (5′-AGC​GGT​GTT​TAT​TTC​CCC​TGG​AAA​TG-3′), 3ctrlFii (5′-AAA​AAA​GGA​TCC​TTA​GAT​CTG​TGA​AAA​ACC​TAA​ATG​ACA​CCA​TCA​CC-3′), and 3ctrlRii (5′-AAA​AAA​GCG​GCC​GCC​TGC​CTC​TCT​CTC​TCA​TAC​ACA​TGT​G-3′).

The human HT-1080–derived cell line containing *tet* operator and *lac* operator arrays, separated by 750 kbp of DNA and expressing TetR-4mCherry and EGFP-LacI was designated TT68 and was created as follows. TT51 (containing *tet* operator arrays and whose construction is described in the previous paragraph) was sequentially transfected with pT2415 and pEGFP-LacI-NLS with selection for histidinol and hygromycin resistance, respectively. These cells were then transfected with pT2838 and pT2847 and blasticidin-resistant clones were obtained. Targeting of the *lac* operator arrays to the desired genomic location was confirmed using the primers 5Fiii (5′-CTC​ATT​ATC​TGT​ACA​TTT​CTT​TGC​ATC​G-3′), 5Riii (5′-CTC​ATC​AAT​GTA​TCT​TAT​CAT​GTC​TGG​ATC-3′), 5ctrlFiii (5′-AAA​AAC​TCG​AGA​TTA​ACT​TCC​ACT​ACT​CTA​CTA​GAG​CTG-3′), 5ctrlRiii (5′-AAA​AAG​GAT​CCT​TGT​CGA​CAT​CTT​GGA​TAC​TAC​CTA​CGT​ATG​TAT​G-3′), 3Fiii (5′-CGT​ATA​ATG​TAT​GCT​ATA​CGA​ACG​GTA​G-3′), 3Riii (5′-CTA​TCA​GAG​ATC​AGT​ACA​AGA​GAG​CAG​TTG-3′), 3ctrlFiii (5′-AAA​AAA​GGA​TCC​TTA​GAT​CTA​CTA​CGG​TGA​AGC​CTA​CAT​AGA​C-3′), and 3ctrlRiii (5′-AAA​AAA​GCG​GCC​GCC​ACT​GTA​TTA​TTT​TCC​TAG​AGC​TGC​CC-3′) using a similar strategy to that shown in Fig. 1 C for integration of the *lac* operator array at an alternative location.

The TT75 derivative expressing Cerulean-PCNA was designated TT104 and was created by transfecting TT75 cells with pmCerulean-PCNA-19-SV40NLS-4 with selection for G418 resistance.

The TT75 derivative with a deletion of the CTCF-binding region close to the *lac* operator array was designated TT108 and was created by transfecting cells with pT3093 and pT3099. Stable clones were obtained and screened for the deletion by PCR using the primer pair CTCF-F (5′-TGC​ATT​TTA​AGT​GCT​CAC​TAG​AGG-3′) and CTCF-R (5′-GTG​CCA​TTC​AGA​ACA​TTT​TTA​GAG-3′). The region around the deletion was sequenced using the primer CTCF-R.

The DT40 cell line containing *lac* operator arrays and *tet* operator arrays was designated TT56 and was a derivative of BM-lacO-20K-19. BM-lacO-20K-19 was a DT40 cell line in which a *lac* operator array (512 repeats) had been targeted to a position 3.8 Mbp along the Z chromosome (Hori et al., 2013; Fig. S2 A). The TT56 cell line was created by transfecting these cells with the plasmid pHHTetO100 and selecting for puromycin resistance. Stable clones were obtained and screened by Southern blotting; genomic DNA was digested with EcoRI and the probe was generated using the primer pair 100South-F (5′-TTT​GCA​GAG​GTC​CAT​GGC​TCC​CCA​ACC​CAG-3′) and 100South-R (5′-GTT​AGC​AAG​CCT​GCA​ATA​TCA​AGA​AAG​GAG-3′). A successfully targeted clone was then transfected with pT2415 and stable clones obtained by selecting for histidinol resistance.

### SDS PAGE and Western blotting

For Western analysis, total cell extracts were obtained from cells grown in 6-well dishes and lysed in 30–60 µl of lysis buffer (20 mM Hepes, pH 7.6; 400 mM NaCl; 1 mM EDTA; 25% glycerol; 0.1% NP-40) containing protease inhibitors (cOmplete EDTA-free; Roche). Lysates were quantified using Bradford reagent (Thermo Fisher Scientific; 1863028) and 30 µg of total protein for each sample was run on precast Bis-Tris 4–12% gradient gels (Invitrogen) and protein transferred to polyvinylidene fluoride membrane (Amersham). Membranes were blocked in PBS containing 5% milk and were incubated with antibodies in PBS containing 2% BSA and 0.05% (wt:vol) sodium azide. Primary antibodies were used as follows: WAPL (Abcam; ab70741), 1 in 5000; RAD21 (Millipore; 05–908), 1 in 2000; NCAPD2 (Sigma; HPA036947), 1 in 1500; NCAPD3 (Bethyl; A300-604A), 1 in 3000; SMC2 (Abcam; ab10412), 1 in 5000; actin (Sigma; A5441), 1 in 20,000. Secondary antibodies were used as follows: donkey-anti-mouse-800CW (LI-COR; 926-32212), 1 in 10,000; donkey-anti-rabbit-680RD (LI-COR; 926–68073), 1 in 10,000. Signal from the secondary antibody was detected using a LI-COR Odyssey CLx.

### Metaphase spreads

After appropriate treatment, ∼2 × 10^6^ cells were collected and incubated in 5 ml of hypotonic solution (75 mM KCl) for 10 min at 37°C. The cells were then incubated in 5 ml of cold fixative (methanol:acetic acid, 3:1) for 20 min at 37°C. This fixation step was repeated and cells finally resuspended in ∼200 µl of fixative solution and stored at −20°C. For spreading, 10–50 µl of fixed cells were dropped onto glass slides and air dried. The spread chromosomes were mounted in Prolong Gold Antifade containing DAPI (Invitrogen) before imaging. In some experiments, the slides were further processed for FISH (for more details see below).

### Generation of FISH probes

FISH probes were designed, prepared, and used essentially according to protocols received from the Bickmore laboratory (Mahy et al., 2002). Specific details are provided below.

Fosmid clones containing ≤40 kb of DNA from the relevant chromosomal regions were identified using the human genome browser at University of California, Santa Cruz, and obtained as *Escherichia coli* stab cultures from http://bacpacresources.org (clones W12-1373D12, W12-1752D18, W12-2889B21, W12-1537E22, W12-819P15, W12-3198E10). They were streaked out onto LB agar plates containing 25 µg/ml chloramphenicol, and cultures were grown from single colonies. Purified fosmid DNAs were obtained from ∼10 ml of overnight culture using a plasmid miniprep kit (Qiagen; 27106). Preparations were quantified using a nanodrop spectrophotometer and confirmed by sequencing at one end using the T7prom primer (5′-TAA​TAC​GAC​TCA​CTA​TAG​GG-3′; fosmids W12-1537E22, W12-819P15, W12-3198E10) or by PCR confirmation using primers specific for the given region (fosmid [primer pairs]: W12-1373D12 [5Chr5-26Mbii-F/5Chr5-26Mbii-R], W12-1752D18 [Chr5-26Mb-5′F/Chr5-26Mb-5′R], W12-2889B21 [5Chr5-26Mbiii-F/5Chr5-26Mbiii-R]).

Fluorescently labeled FISH probes were generated from 1 µg of fosmid DNA using a nick translation kit (ENZO; ENZ-GEN111-0050) and the 5-fluorescein (ENZO; ENZ-42831; for probe 3; green) or ATTO-647 (JENA Biosciences; NU-803-647N-S; for probes 1, 2, 4, 5 and 6; red)–labeled dUTPs according to the manufacturer’s instructions. After labeling, 100 ng of probe was mixed with 6 µg of human Cot1 DNA (ENZO; ENZ GEN116-0500), containing enriched human repetitive genome DNA sequences, and 5 µg of salmon sperm DNA (Sigma). The FISH probe mixture was then ethanol precipitated, dried, and stored at −20°C ready for FISH hybridization procedure.

### FISH in methanol-acetic acid–fixed cells

Cells were fixed and dropped onto glass slides as though for metaphase spreads (see above procedure). However, for FISH, after dropping the cells onto slides they were left at room temperature for 2–3 d. Before hybridization to probes, the slides were incubated in 2× SSC buffer containing 100 µg/ml RNase A (Invitrogen) at 37°C for 1 h. Slides were washed briefly in 2× SSC and then dehydrated through sequential 2-min incubations in 70%, 90%, and 100% ethanol before air-drying the slides. Denaturation buffer (70% deionized formamide, 2× SSC, pH 7.5) was prepared fresh and warmed to 70°C, and then the fixed cells were denatured at 70°C for 3 min. The slides were then quickly transferred to ice-cold 70% ethanol for 2 min and then dehydrated through sequential 2-min incubations in 90% and 100% ethanol. The slides were then dried and stored at room temperature while probes were prepared for hybridization.

Single aliquots of FISH probe mixture, which had been prepared and stored at −20°C previously (see above), were dissolved for 1 h at room temperature in 30 µl hybridization buffer (50% deionized formamide, 2× SSC, 10% dextran sulfate, 1% Tween20). They were then denatured at 70°C for 5 min before placing at 37°C to preanneal for 15 min. A total volume of 30 µl containing dissolved probes (e.g., 15 µl of probe “a” mixture plus 15 µl of probe “b” mixture) was then placed onto 37°C prewarmed slides containing the cell spreads and covered and sealed under a 15-mm coverslip and incubated at 37°C overnight.

After hybridization, slides were washed four times for 3 min with 2× SSC at 45°C and then four times for 3 min with 0.1× SSC at 60°C. The slides were briefly washed in 4× SSC containing 0.1% Tween20 and then mounted using 25 µl of prolong gold antifade containing DAPI (Invitrogen; P36935), which was allowed to polymerize overnight at room temperature before image acquisition by microscopy.

### ChIP

Cells were plated and grown in 10-cm dishes until confluent (1–2 × 10^6^ cells). For each ChIP, ∼4 × 10^6^ cells were used (2 × 10-cm dishes). Cells were cross-linked with 1.42% (vol:vol) formaldehyde for 10 min and quenched with 125 mM glycine for a further 10 min. Cells were washed three times in 10 ml PBS and then scraped from the dishes. Cells were resuspended in 2 × 300 µl lysis buffer containing 1% SDS, 10 mM EDTA, 50 mM Tris, pH 8.1, and protease inhibitors (cOmplete EDTA-free; Roche); and chromatin was fragmented (to ∼0.5 to 1.5 kb) using a Biorupter (Diagenode) at medium setting with total sonication time of 10 min (20 cycles of 30 s on/off). Lysates were cleared by centrifugation for 10 min at high speed, and 50 µl was saved to create the input sample. The remaining lysates were diluted 1:10 in ChIP dilution buffer containing 1% Triton X-100, 2 mM EDTA, 150 mM NaCl, 20 mM Tris, pH 8.1, and 0.1% Brij-35. The lysates were then incubated for 1 h at 4°C with 60 µl Dynabeads Protein G beads or Dynabeads for pan IgG mouse (both Invitrogen). Beads were then removed, and these lysates were incubated with 2 µg of antibody (see below) overnight at 4°C. 60 µl of Protein G Dynabeads for antibodies from rabbit or pan-IgG-mouse Dynabeads for antibodies from mouse that had been incubated overnight in PBS containing 0.5% (wt:vol) BSA were then added to the lysate plus antibody mix and incubated for a further 4 h. The supernatant was then discarded and the beads washed twice with 1 ml of each of the following; wash buffer I (0.1% SDS, 1% Triton X-100, 2 mM EDTA, 20 mM Tris, pH 8.1, and 150 mM NaCl), wash buffer II (0.1% SDS, 1% Triton X-100, 2 mM EDTA, 20 mM Tris, pH 8.1, and 500 mM NaCl), wash buffer III (0.25 M LiCl, 1% NP-40, 1% sodium deoxycholate, 1 mM EDTA, and 10 mM Tris, pH 8.1), and TE buffer (10 mM Tris, pH 8.1, and 1 mM EDTA). Antibody-protein-DNA complexes were then eluted in 2 × 100 µl of elution buffer (1% SDS and 0.1 M sodium bicarbonate). Cross-links were then reversed for these samples and the input sample by adding 8 µl 5-M NaCl and incubating at 65°C overnight. Next 10 µl of RNaseA (10 mg/ml) was added and incubated at 37°C for 30 min and then 4 µl 0.5-M EDTA, 8 µl 1-M Tris, pH 8.1, and 10 µl proteinase K (15 mg/ml) were added and incubated at 45°C for 2 h. Finally, DNA was purified using minElute DNA purification columns (Qiagen) and eluted in 20 µl of elution buffer. Antibodies used for ChIP were anti-CTCF and mouse-IgG (both Millipore; 17-10044), anti-SMC3 (Abcam; ab9263), and rabbit-IgG (Sigma). Mouse IgG and rabbit IgG were used for ChIP control.

### qPCR

qPCR was performed using an Eppendorf LightCycler 96 and RotorGene SYBRGreen PCR mix (Qiagen; 1054596) according to the manufacturer’s instructions. For each experiment, technical replicates were prepared for each sample. Input chromatin was diluted 1:100. To compare the overall pull-down efficiency between cell lines, the following positive-control primers were used: CTCF-pos-F (5′-CGG​AGT​ATC​AAG​GCA​TCA​GTA​A-3′) and CTCF-pos_R (5′-GGA​ATC​GCA​CAG​TTG​AGA​ATA​AG-3′). To assess pull-down of our region of interest, two sets of query primers were used CTCF-qu-F1 (5′-GTC​ATG​TCT​TCA​GTG​CAT​GAT​TT-3′), CTCF-qu-R1 (5′-GGT​AGG​GAC​TAA​GTC​TGT​TTC​G-3′) and CTCF-qu-F2 (5′-AGT​GTC​ATT​AGT​GCT​TCC​TTC​T-3′), CTCF-qu-R2 (5′-GAG​AAT​GCT​CTG​GCC​TCT​TT-3′). After PCR, ΔCq values for each sample were obtained by Cq_input_ – Cq_pull-down_; from this, we calculated each as a fraction of input (2^−ΔCq^) to measure pull-down efficiency. Finally, each value was normalized as a fraction of the pull-down achieved for the positive control primers on chromatin from the WT cells.

### Live-cell microscopy and image analysis

Time-lapse images were collected at 37°C with 5% CO_2_ using a DeltaVision ELITE microscope (Applied Precision). We used an apochromatic 100× objective lens (Olympus; numerical aperture: 1.40) to minimize longitudinal chromatic aberration. We also regularly checked lateral and longitudinal chromatic aberration using 100-nm multi-color beads. We did not detect any chromatic aberration between the colors observed in the current study.

For signal detection we used either a CoolSNAP HQ or EMCCD Cascade II camera (both Photometrics). For HT-1080 derivative cells, we acquired 25 z-sections 0.75 µm apart. For DT40 cells, we acquired 25 z-sections 0.5 µm apart. During live-cell imaging, Cerulean, EGFP, and mCherry signals were discriminated using the dichroic CFP/YFP/mCherry (52-850470-000 from API). For imaging chromosomes in live cells, SiR-DNA (Tebu-bio; emission 674 nm) and EGFP signals were discriminated using the dichroic DAPI/FITC/TRITC/Cy5 (52-852111-001 from API).

After acquisition, images were deconvolved using softWoRx software with enhanced ratio and 10 iterations. Analysis of individual cells was performed using Imaris software (Bitplane). Statistical analyses were performed using Graphpad Prism 6.0 software.

The configuration of the fluorescent *tet* and *lac* operator arrays in live cells was determined through time and in three dimensions using Imaris imaging software (Bitplane). In our time-lapse imaging, we use an oil-based objective while our cells were grown in a water-based medium. To correct for the refractive index mismatch, we multiplied the assigned z distance of 0.75 µm by a scaling factor of 0.85, which gives ∼0.64 µm per z step (Besseling et al., 2015). For *tetO-tetO*, *lacO-lacO*, and *tetO-lacO* distance measurement in Fig. S1, D and E, Imaris was used to automatically assign xyz coordinates for the center of mass of the fluorescent spots (the centroid) and these were used to calculate the distance between them. When Imaris could not reasonably identify centroids (∼26% of cases) when background signal was high, we manually obtained coordinates for the center of mass using the image inspector tool. Our assignment of dot configurations into categories (or states) is based on measurements in 3D using the following rules: For both blue “nonresolved” and brown “partially resolved” states, at least one operator (*tetO* or *lacO*) was observed as a single fluorescent object. To distinguish blue “nonresolved” and brown “partially resolved” states, we looked at the second operator region such that (a) if it was observed as a single fluorescent object or appeared as two fluorescent objects whose centers were separated by <0.85 µm, we classified it as the blue “nonresolved” state, or (b) if it was observed as two fluorescent objects whose centers were separated by >0.85 µm, we classified it as the brown “partially resolved” state. For both pink “resolved” and red “nonresolved” states, both of the operator regions appeared as two separate fluorescent objects (four objects in total; two for *lacO* and two for *tetO*). To distinguish pink “resolved” and red “nonresolved” states, we looked at the colocalization of the *lacO/tetO* paired fluorescent objects, such that (a) if one pair (or both) showed <50% colocalization, we classified them as the pink “partially resolved” state, or (b) if both pairs showed >50% colocalization, we classified them as the red “compacted” state. In some cases, following NEBD, all four fluorescent dots showed >50% colocalization over ≥5 consecutive time points and we classified this as a black “nonresolved and compacted” state. White spaces in Fig. 2 B, Fig. S2 K, Fig. S3 G, Fig. S4 B, and Fig. S5 A represent time points where configuration could not be determined. To plot the proportions of color-coded configurations of the fluorescence reporter over time (Figs. 2 D, 3 E, 4 F, 6 B, 7 C, 8 G, S2 H, and S4 C), smoothing was applied by calculating the rolling mean proportion across 9 min.

Using Imaris imaging software (Bitplane) we determined the time of transition between the end of S phase and the start of G2 by observing the behavior of Cerulean-tagged PCNA—a component of the replication machinery. For the first time point of each movie we used a semiautomatic approach to define the nuclear PCNA dots that correspond to replication factories (Imaris detects spots according to a user-defined threshold). Imaris then automatically tracked the fate of these dots through space and time (allowing maximum movement of 5 µm between time points and only connecting those dots that have no more than two time-point gaps). Fluorescent dots that did not fall in these tracks, or tracks of ≥30 min, were then excluded as random background signal. We then defined the end of S phase for individual cells as the time when all the Imaris-detected Cerulean-PCNA dots had disappeared.

Using Imaris imaging software (Bitplane) we determined the average length of prophase by automatically measuring the changes in chromosome volume, as a measure of compaction status, over time by visualization of the DNA using the live-DNA stain SiR-DNA. We aligned these movies relative to NEBD that was defined as the time at which EGFP-LacI-NLS redistributes from the nucleus to the whole cell. For the first time point of each movie, we used a semiautomatic approach to accurately define the chromosomal DNA of the nucleus (Imaris defines a surface corresponding to the far-red fluorescence of the SiR-DNA signal using background subtraction according to a user-defined threshold). Imaris then automatically detected and tracked the chromosomes, across subsequent time points during which mitotic chromosome organization, NEBD, and anaphase occurred. At anaphase, only one set of the divided chromosomes was tracked further. For individual cells, the volume of the chromosomes and their average mean intensity at each time point were normalized to those at NEBD. A mean volume and mean intensity for all the cells were then plotted, and the time when DNA volume began to decrease was defined as the start of prophase.

### Fixed-cell microscopy and image analysis

Images of fixed cell were collected using the DeltaVision ELITE microscope setup mentioned above. For metaphase spreads, 15 sections (0.2-µm interval) were acquired for simple DAPI staining or 10 sections (0.5-µm interval) for three-color FISH analysis. DAPI, ATTO-647, and 5-fluorescein signals were detected using the dichroic DAPI/FITC/TRITC/Cy5 (52-852111-001 from API) and an EMCCD Cascade II camera.

After acquisition, images were deconvolved using softWoRx software with enhanced ratio and 10 iterations. Images were analyzed using Imaris software (Bitplane).

For the FISH experiments in prophase, in a similar way to the live-cell imaging we defined the configuration of individual pairs/sets of fluorescent dots (hybridized FISH probes) by assigning them into nonresolved (blue), partially resolved (brown), or resolved (pink) states. Since preparation of cells for FISH made them flat, we considered the configuration of fluorescent dots in two dimensions in projected images. We excluded metaphase chromosomes (those containing individualized and highly condensed chromosomes) from this analysis since our focus was prophase. In some images, punctate background signals (green or far-red) were observed. To avoid such background signals, we only considered clusters of signals that showed both green and far-red signals in close proximity (<1.5 µm); since all pairs of FISH probes locate within 1 Mbp on genome, their signals should be in close proximity if they are properly hybridized on genome DNA.

### ENCODE datasets and analyses

ENCODE datasets (ENCODE Project Consortium, 2012) were searched and displayed using the genome browser at University of California, Santa Cruz (http://genome.ucsc.edu/) with the Human Feb. 2009 (GRCh37/hg19) Assembly (Kent et al., 2002). The ENCODE Genome Expression Omnibus (GEO) accession numbers were as follows: GSM935542 (SMC3; Bernstein laboratory, Harvard University, Cambridge, MA), GSM935647 (RAD21; Snyder laboratory, Stanford University, Stanford, CA), and GSM733645 (CTCF; Bernstein laboratory).

To identify sites of cohesin enrichment in ChIP-seq datasets, we analyzed ENCODE datasets using the Galaxy Web platform on the public server at usegalaxy.org (Afgan et al., 2018). We selected data obtained from HEP-G2 cells since they are near-diploid (modal chromosome number 55) and therefore have similar karyotype to diploid HT-1080 cells. The ENCODE GEO accession numbers of raw ChIP DNA sequence data used are as follows: ENCFF000XXY/ENCFF000XYC (SMC3; Snyder laboratory, Stanford); ENCFF000XXK/ENCFF000XXL (RAD21; Snyder laboratory; ENCODE Project Consortium, 2012). The datasets were mapped to the human genome (hg19), using the Bowtie2 function, and filtered using Samtools. Subsequently, significant peaks of enrichment were identified using the MACS2 algorithm (Galaxy version 2.1.1.20160309.4; Zhang et al., 2008; Feng et al., 2012) with m-fold limits of 5–50, bandwidth of 300, and a minimum FDR (q-value) cutoff of 0.01.

### Mathematical modeling

We developed a computer model that describes both the transition of a marked chromosomal region to the resolved configuration and the subsequent transition to a compacted state (Fig. 8, A and B). The model is calculated over multiple cells.

A cell *i* is a sequence of states *S_i_*(*t_k_*) on a discrete time grid *t_k_* built to match the extent of time points of microscopy data. We use *t_k_* from −140 to 90 min, with a constant time step of 1 min *t_k_* = 0 corresponds to NEBD. The states *S_i_*(*t_k_*) are assigned in an iterative process over *k* = 1, . . . , *n* (here *n* = 231), based on the current state and certain state transition rules. The transition from “unresolved” to “resolved” state can happen only after a licensing time ${\hat{t}}_{1}$, with a certain probability. The transition from “resolved” to “compacted” state can occur only after another licensing time ${\hat{t}}_{2}$, with a certain probability. The licensing times and probability rules for state switching are as follows.

The model is based on Poisson processes. For the given cell, the licensing times are randomly drawn from exponential distributions. Specifically, ${\hat{t}}_{1}$ takes place before NEBD, ${\hat{t}}_{1}=t_{NEBD}-R_{e}(\tau_{1})$ and ${\hat{t}}_{2}$ happens after ${\hat{t}}_{1}$, and ${\hat{t}}_{2}={\hat{t}}_{1}+R_{e}(\tau_{2})$, where $R_{e}(\tau )$ is a random variable with cumulative distribution $1-e^{-t/\tau }$. In the time coordinates defined here, *t_NEBD_* = 0.

Once the licensing times are generated, the states *S_i_*(*t_k_*) can be computed. The simulation iterates over *k* = 1, . . . , *n*, filling *S_i_*(*t_k_*) with appropriate states. All *S_i_*(*t_k_*) for $t_{k}<{\hat{t}}_{1}$ are filled with “unresolved” state. After ${\hat{t}}_{1}$, the transition from the “unresolved” to the “resolved” state is allowed and occurs at a constant rate *r*_1_. The probability of this transition per unit time Δ*t* is $p_{1}=1-e^{-r_{1}\Delta t}$. At every iteration step, a uniform random variable *R_u_* is compared with *p*_1_. A condition $R_{u}<p_{1}$ indicates a state transition and the following elements of *S_i_*(*t_k_*) are filled with “resolved” state.

After the transition, the iteration continues until $t_{k}\geq {\hat{t}}_{2}$, after which the transition to the “compact” state is allowed and occurs at a rate *r*_2_ with probability $p_{2}=1-e^{-r_{2}\Delta t}$ per time step. This state transition is generated in the same way as the “unresolved” to “resolved” one using a condition $R_{u}<p_{2}$, and the subsequent elements of *S_i_* are filled with “compacted” state. At the end of the iteration, all elements of *S_i_* have an assigned state.

The calculation was repeated over *m* = 10,000 cells and proportions of each state at each time point are found, $P\left(x;\;t_{k}\right)=\left|\left\{i:S_{i}\left(t_{k}\right)=x\right\}\right|/\;m$, where *x* denotes a state. The model is controlled by four parameters, time scales *τ*_1_ and *τ*_2_ and transition rates *r*_1_ and *r*_2_. The algorithm for the model is presented as pseudocode in Fig. S5 E.

The model was fitted to microscopy data using the Broyden–Fletcher–Goldfarb–Shanno algorithm implemented in *optim* function in R package (version 3.4.3). Using this algorithm, we obtained best-fitting parameter values that minimize the sum of squares of differences between the model and microscopy data in the time window from −50 to +30 min relative to *t_NEDB_*.

To assess the uncertainty in fitting parameter values, bootstrapping was performed. From a given microscopy dataset, the data points were randomly sampled (the same number as in the original dataset) with replacement within the above time window (−50 to +30 min); this generated a dataset where some points were missing and some duplicated with respect to the original microscopy dataset. Our model was fitted to the bootstrap dataset and best-fitting parameter values were found. This procedure was repeated 300 times, giving a distribution of values for each parameter (Fig. 8, D and E; and Fig. S5 D). The median of this distribution represents the central value of the parameter (Fig. 8 E).

In box plots (Fig. 8 E), the box indicates the value from the first to third quartile (interquartile range [IQR]) and a thick line in the box shows a median. The upper whisker and lower whisker show the maximum and minimum values, respectively, which do not exceed 1.5 IQR beyond the box. Outliers, which exceed the range between whiskers, are shown as individual data points.

Parameters *τ*_1_ and *τ*_2_ can be understood as typical time scales at which state transitions are licensed. For example, for untreated cells we find *τ*_1_ = 18.0 min. The corresponding half-life time (*τ*_1_ ln2 = 12.5 min) tells us that half of the cells are licensed for transition to the “resolved” state (licensing 1) 12.5 min before NEBD. Similarly, for untreated cells we find *τ*_2_ = 9.3 min. The corresponding half-life time (*τ*_2_ ln2 = 6.5 min) shows that half of the cells are licensed for transition to the “compacted” state (licensing 2) 6.5 min after licensing 1. For simplicity of interpretation, we converted *τ*_1_ and *τ*_2_ values to half-life values in our figures and report them as ST and TD, respectively. Similarly, the rates *r*_1_ and *r*_2_ can be interpreted in terms of half-lives

$$\left(\frac{1}{r}\ln2\right)$$

of decay into a transformed state or as constant probabilities of transition in a single time step, $\mathrm{Pr}\left(r;\Delta t\right)=1-e^{-r/\Delta t}$. For example, *r*_1_ = 0.065 min^−1^ for untreated cells. This corresponds to a decay half-life of 10.7 min or the probability of transition in a single 1-min time step of 0.063.

It should be noted that ST and TD represent typical time (median time) of licensing for transition to the pink “resolved” state (licensing 1) and to the red “compacted” state (licensing 2), respectively, but they do not directly define transition times. Times of transition to the pink “resolved” state and to the red “compacted” state are directly defined by the rates *r*_1_ and *r*_2_, respectively, in cells licensed for each transition (Fig. 8 B). Also note that the transition to the pink “resolved” state can happen after licensing 2 for a fraction of cells; in these cells, transition to the red “compacted” state can only occur after the transition to the pink “resolved” state.

The model was implemented in R. The software with documentation (R markdown document) is available from GitHub at https://github.com/bartongroup/MG_ChromCom.

### Online supplemental material

Fig. S1 shows observation of fluorescent dots in 3D (A), evaluation of the timing of NEBD (B), and measurement of distances between fluorescent dots (C–F). Fig. S2 shows use of the fluorescence reporter system in DT40 cells (A–D), evaluation of timing of the S phase/G2 phase transition and the G2 phase/prophase transition (E–H), the procedure of siRNA treatment and release from a double thymidine block (I), and supplemental results of control, RAD21, and WAPL siRNA treatment (J–L). Fig. S3 shows comparison of two cell lines carrying *lacO* at different chromosome sites (A and B) and supplemental results of deletion of CTCF-binding sites (C–H). Fig. S4 shows the procedure of ICRF-193 treatment (A), supplemental results of ICRF-193 treatment (B–D), the procedure of cell synchronization using RO-3306 treatment and subsequent washout (E), and results of ICRF-193 treatment after this procedure (F and G). Fig. S5 shows supplemental microscopy results of control, NCAPD2, NCAPD3, and SMC2 siRNA treatments (A and B); analyses of these microscopy results using mathematical modeling (C and D); and the pseudocode used in the mathematical modeling (E).

## Supplementary Material

Supplemental Materials (PDF)
