# Supplementary material for: Live imaging of marked chromosome regions reveals their dynamic resolution and compaction in mitosis
Source: J Cell Biol. 2019 Mar 11;218(5):1531–52. doi: 10.1083/jcb.201807125 (PMC6504890; doi:10.1083/jcb.201807125)
Supplement: Supplemental Materials (PDF) [file JCB_201807125_sm.pdf]

JCB

Supplemental material

Eykelenboom et al., https://doi.org/10.1083/jcb.201807125

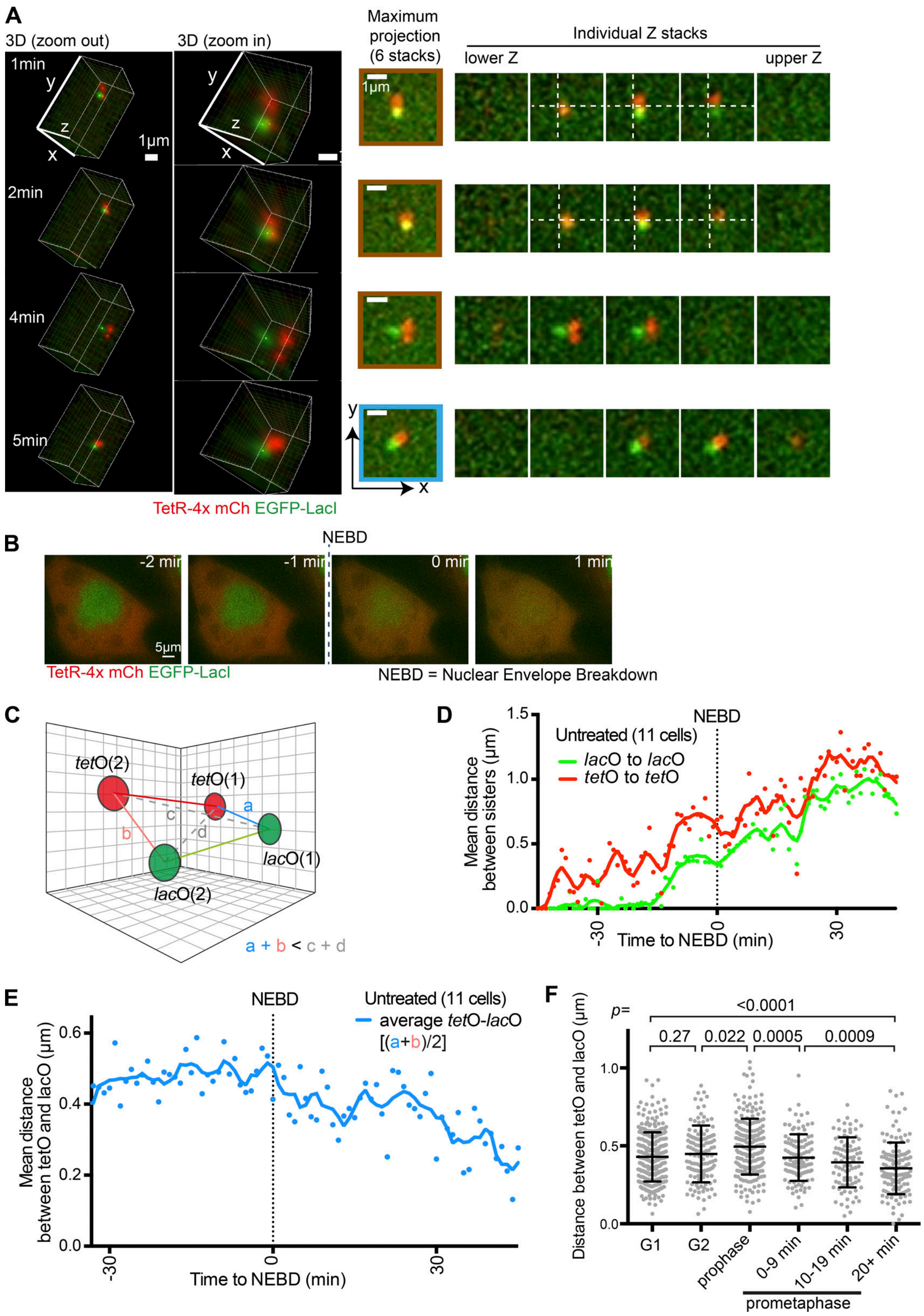
A
3D (zoom out)
3D (zoom in)
Maximum projection (6 stacks)
Individual Z stacks
lower Z
upper Z
1min
2min
4min
5min
1µm
TetR-4x mCh EGFP-LacI
B
NEBD
-2 min
-1 min
0 min
1 min
5µm
TetR-4x mCh EGFP-LacI
NEBD = Nuclear Envelope Breakdown
C
tetO(2)
tetO(1)
lacO(1)
lacO(2)
a + b < c + d
D
NEBD
Untreated (11 cells)
lacO to lacO
tetO to tetO
Mean distance between sisters (µm)
Time to NEBD (min)
E
NEBD
Untreated (11 cells)
average tetO-lacO [(a+b)/2]
Mean distance between tetO and lacO (µm)
Time to NEBD (min)
F
p=
<0.0001
0.27
0.022
0.0005
0.0009
Distance between tetO and lacO (µm)
G1
G2
prophase
0-9 min
10-19 min
20+ min
prometaphase

Figure S1. **3D visualization and distance analyses of *tetO* and *lacO* arrays. (A)** An example of 3D analysis of fluorescent dots. Matched 3D (left), maximum projection (middle) and individual z-stack (right) view of fluorescent dots, observed in an individual cell over time (top to bottom). In 3D view (zoom out), the fluorescent dots change their locations over time in the selected and fixed 3D microscopy space. In 3D view (zoom in), fluorescent dots are zoomed in. The individual z-stack only shows the consecutive z-sections containing the fluorescent dots (plus one or two). The configuration of the fluorescent dots at each time point was determined by their observation of the individual fluorescent dots in 3D (x, y, z) and indicated with color frames (colors as in Fig. 2, A and B) on maximum projection views. The 3D analysis was necessary for two reasons: (1) in some cases, dot separation occurred along the *z* axis, which could be determined by scanning through the z-stack images (right-hand side) as well as by using the "spot detection" tool on Imaris in the 3D space; (2) to ensure that all fluorescent dots had been included to accurately determine the configuration (note the lack of detection of the specific fluorescent signal in the upper and lower z-stacks). Times indicated are relative to the beginning of the time lapse. Small solid red and green points indicated on the 3D views (left) are the center of mass (centroid) of the fluorescent dots determined using Imaris. White dotted lines are drawn at a fixed x-y location in selected images to accentuate the offset in the xy dimensions. **(B)** Time-lapse images of an individual cell undergoing NEBD. TT75 cells were arrested by a double-thymidine block and subsequently released from the block. 8–10 h after the release, images were taken every minute. Times indicated are relative to NEBD. Scale bar, 5 µm. **(C)** Diagram representation of the *tetO* and *lacO* and the distance relationships between them in a 3D volume. We analyzed the distances between sister arrays (i.e., *tetO*-*tetO* or *lacO*-*lacO*) and the pair of *tetO*-*lacO* distances; for the latter, we chose "a" and "b" for *tetO*-*lacO* distances, rather than "c" and "d," when a + b was smaller than c + d. **(D)** Graph showing average distance between sister chromatids as measured using the *tetO*-*tetO* and *lacO*-*lacO* distances plotted against time to NEBD. Distance measurements were obtained from individual cells and aligned according to NEBD. At each time point the mean distance between sister *tetO* or *lacO* among cells was plotted (red and green points). The corresponding red (*tetO*-*tetO*) and green (*lacO*-*lacO*) lines are smoothed averages with second-order smoothing (four neighbors). The measurements were obtained from the 11 untreated cells at the top of Fig. 2 C. **(E)** Graph showing average distance between *tetO* and *lacO* plotted against time. Distance measurements were obtained from individual cells and aligned according to NEBD. At each time point relative to NEBD, the mean distance between *tetO* and *lacO* (the average of "a" and "b" shown in part C) among cells was plotted (blue points). The corresponding blue line is a smoothed average with second-order smoothing (4 neighbors). The measurements were obtained from the 11 untreated cells at the top of Fig. 2 C. **(F)** Graph showing individual *tetO*-*lacO* distances ([a + b]/2; see part C) plotted for specific cell-cycle time periods. Measurements for G1 cells were obtained by time-lapse imaging of 10 cells that had been arrested in G1 using a double-thymidine block. Measurements made during the time period up to 20 min before NEBD were classified as G2 and those during the last 20 min before NEBD were classified as prophase (see also Fig. S2 G). Measurements made during the time period after NEBD were classified as prometaphase. The G2, prophase, and prometaphase measurements were obtained from the 11 untreated cells at the top of Fig. 2 C. The mean and SD for each condition is shown. P values for difference between time periods were obtained by unpaired *t* tests. The number of analyzed points for G1, G2, and prophase were 306, 129, and 213, respectively. The number of analyzed points for the three prometaphase subcategories (0–9, 10–19, and ≥20 mins) were 109, 88, and 132, respectively. Reflecting dynamic behaviors of fluorescent dots, variation of the dot distance was relatively large at each phase. The mean distance slightly increased from G2 to prophase and then decreased from prophase to prometaphase. The slight increase in prophase may reflect transient chromosome expansion (Liang et al., 2015) or dissolution of chromatin loops that occurs at mitotic entry (Gibcus et al., 2018). The mean *tetO*-*lacO* distance was reduced from prophase to prometaphase and during prometaphase progression, which is consistent with the coincidental increase of the red "compacted" state (Fig. 2 D).

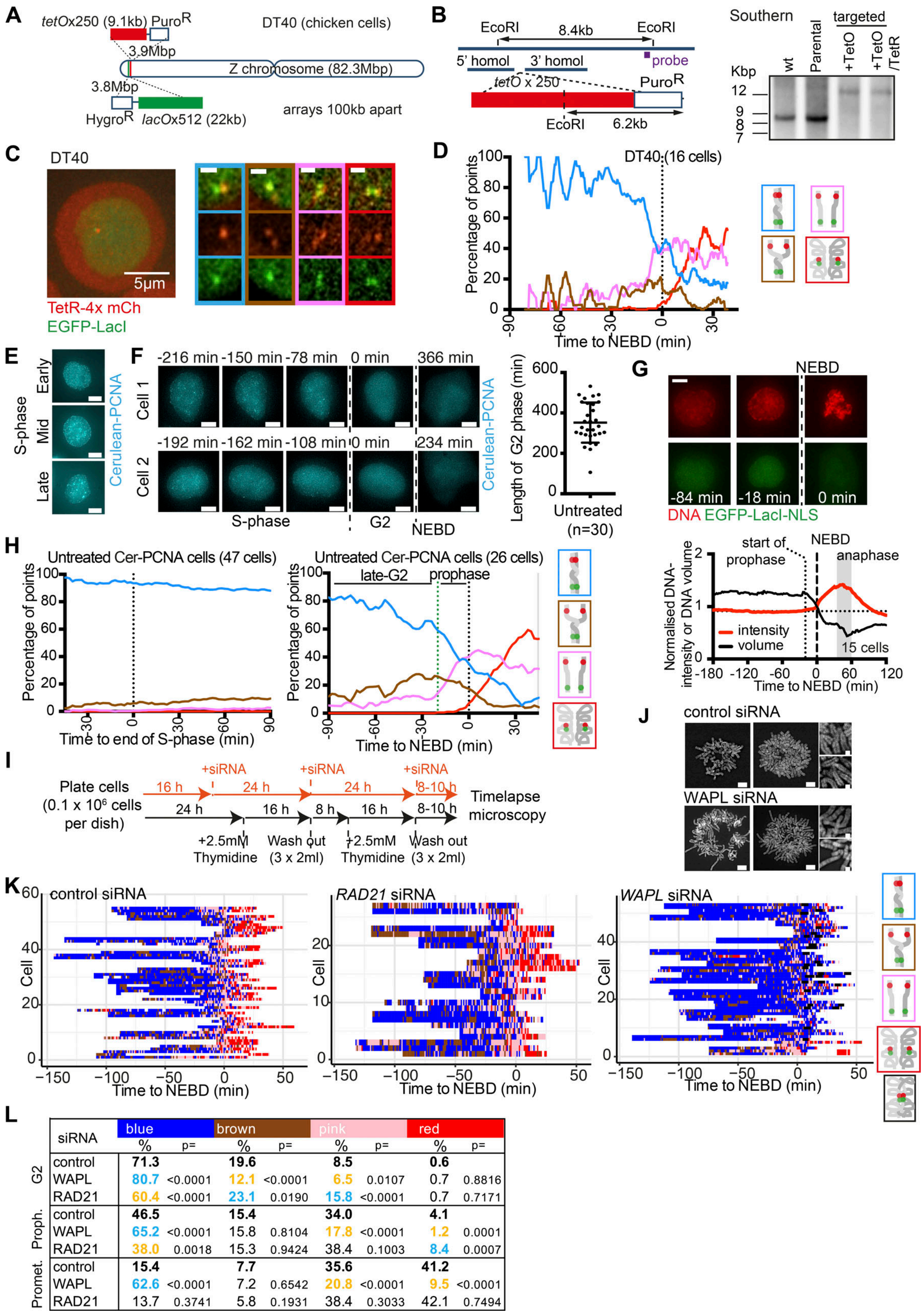

L

	siRNA	blue %	p=	brown %	p=	pink %	p=	red %	p=
G2	control	**71.3**		**19.6**		**8.5**		**0.6**	
	WAPL	80.7	<0.0001	12.1	<0.0001	6.5	0.0107	0.7	0.8816
	RAD21	60.4	<0.0001	23.1	0.0190	15.8	<0.0001	0.7	0.7171
Proph.	control	**46.5**		**15.4**		**34.0**		**4.1**	
	WAPL	65.2	<0.0001	15.8	0.8104	17.8	<0.0001	1.2	0.0001
	RAD21	38.0	0.0018	15.3	0.9424	38.4	0.1003	8.4	0.0007
Promet.	control	**15.4**		**7.7**		**35.6**		**41.2**	
	WAPL	62.6	<0.0001	7.2	0.6542	20.8	<0.0001	9.5	<0.0001
	RAD21	13.7	0.3741	5.8	0.1931	38.4	0.3033	42.1	0.7494

Figure S2. **Analyses of *tetO* and *lacO* arrays in chicken DT40 cells, during different cell cycle stages in human cells, and following depletion of RAD21 or WAPL in human cells. (A)** Diagram depicting the location of *tetO* and *lacO* introduced into the Z chromosome of DT40 cells using homologous recombination gene-targeting methods. **(B)** The map (left) shows the targeting strategy for integration of *tetO* to the region of the Z chromosome shown in A. The map also shows relative positions of EcoRI restriction sites in the genome (dark blue line, top) or the *tet* operator array region (red box, bottom) and the position of the DNA probe (purple line) for the Southern blot screen. Southern blotting (right) using the indicated probe reveals a 12-kb product for the targeted clones and an 8.4-kb product for the WT cells. **(C)** Visualization of these arrays by expression of TetR-4x mCherry and EGFP-LacI in DT40 cells (TT56) (left). Scale bar, 5 µm. Representative images of the major configurations of the fluorescent reporter observed in TT56 cells (right). Designated color codes for each are indicated in the image frames. Scale bar, 1 µm. **(D)** Behavior of the fluorescence reporter observed in WT live cells. Asynchronously growing TT56 cells were imaged every 30 s and, at each time point, the configuration of the reporter was determined and represented using the color codes depicted in C. Data from individual cells were aligned according to the occurrence of NEBD (time zero), and proportions of each configuration at each time point were determined with smoothing applied by calculating the rolling mean proportion across 9 min. Color coding for each configuration is indicated on the right-hand side. **(E)** Snapshot images of Cerulean-PCNA observed in live asynchronously growing TT104 cells. Images show replicating cells with characteristic PCNA foci state of early, mid, and late S phase. Scale bar, 10 µm. **(F)** Measuring the length of G2 phase in individual TT104 cells by observing Cerulean-PCNA behavior. TT104 cells were arrested by a double-thymidine block and subsequently released from the block. 5 h after the release, images were taken every 6 min. On the left, time-lapse images of Cerulean-PCNA are shown, and times indicated are relative to the point of disappearance of the Cerulean-PCNA foci as determined using Imaris software (end of S phase). NEBD was identified after the diffusion of free Cerulean-PCNA throughout the cell. The phases of the cell cycle of the images are indicated. Scale bars, 10 µm. The G2 phase time period for individual cells (between the end of S phase and NEBD) was measured and plotted (right). **(G)** The average length of prophase for TT75 cells was determined as the time elapsed between the onset of chromosome compaction and NEBD. TT75 cells were incubated with SiR-DNA and their images were acquired every 2 min. A representative cell is shown at the top. Timing of NEBD was defined as the time of dispersion of GFP-LacI signal from the nucleus to the whole cell (time zero). For individual cells, the total volume of the chromosomes was determined at each time point using Imaris imaging software, and this was used to assess the degree of chromosome compaction. Measurements made in individual cells were normalized to the volume of the cell in question at NEBD. At each time point relative to NEBD, the mean normalized volume among cells was plotted (black line on graph). When a decrease in chromosome volume was observed, there was a corresponding increase in average signal intensity within the chromosome volume. The red line shows the mean normalized signal intensity of all cells plotted against time to NEBD. Finally, two further predictable drops in DNA volume (for individual cells or cell average) after NEBD were observed corresponding to continuing chromosome compaction during prometaphase and onset of anaphase (whose timing across the population is indicated by a shaded gray bar; Mora-Bermúdez et al., 2007) (note that we only track the volume for one of the sets of dividing chromosomes), providing validation that our method of measuring chromosome volume can provide an accurate picture of the processes of chromosome organization during mitosis. The chromosome volume starts reduction at −20 min relative to NEBD, so we define this time as the prophase onset (indicated by a dotted line). **(H)** Behavior of the fluorescence reporter observed in WT live cells. TT104 cells were synchronized as in F. 5 h (late S and early G2) or 8–10 h (late G2 and prophase) after the release images were taken every 2 min and, at each time point, the configuration of the reporter was determined and represented using the color codes depicted on the right. Data from individual cells were aligned according to the end of S phase as determined according to F (left plot) or according to NEBD (right plot) and proportions of each configuration at each time point were determined with smoothing applied by calculating the rolling mean proportion across nine time points. **(I)** Experimental procedure outline. siRNA was added to cells 16 h after plating and then cells were synchronized in early S phase using a double-thymidine block. After each thymidine washout the relevant siRNA was added back to the cells as indicated. **(J)** Spread of metaphase chromosomes using WAPL-depleted and control cells. Asynchronously growing TT75 cells were treated with WAPL or control siRNA for 48 h before fixation and metaphase spread preparation. Shown are representative images obtained. Scale bars, 5 µm (large images; left) or 1 µm (zoomed images; right). **(K)** Behavior of the fluorescence reporter as observed in individual live cells, treated with control, RAD21, or WAPL siRNA (respectively), plotted across the *y* axis. TT75 cells were treated with siRNA, arrested by a double-thymidine block, and subsequently released from the block. 8–10 h after the release, images were taken every minute and, at each time point, the configuration of the reporter was determined and represented using the color codes depicted in Fig. 2, A and B. Data from individual cells were aligned as in Fig. 2 C. White spaces represent time points where configuration could not be determined. **(L)** Table showing systematic analyses comparing the frequency of each conformation (nonresolved, blue; partially resolved, brown; resolved, pink; compacted, red) for each siRNA knockdown condition (WAPL or RAD21) with the frequency seen under control siRNA conditions during either G2 phase (−90 to −21 min), prophase (−20 to −1 min), and prometaphase (0 to 45 min). Percentages for the control conditions are shown in bold and black. The percentage for each test condition is shown along with the P value calculated using a chi-square test. Where there is a statistically significant increase or decrease in frequency under the test conditions, the percentage is indicated in blue or orange, respectively. The data were taken from K. Cer-PCNA, Cerulean-PCNA.

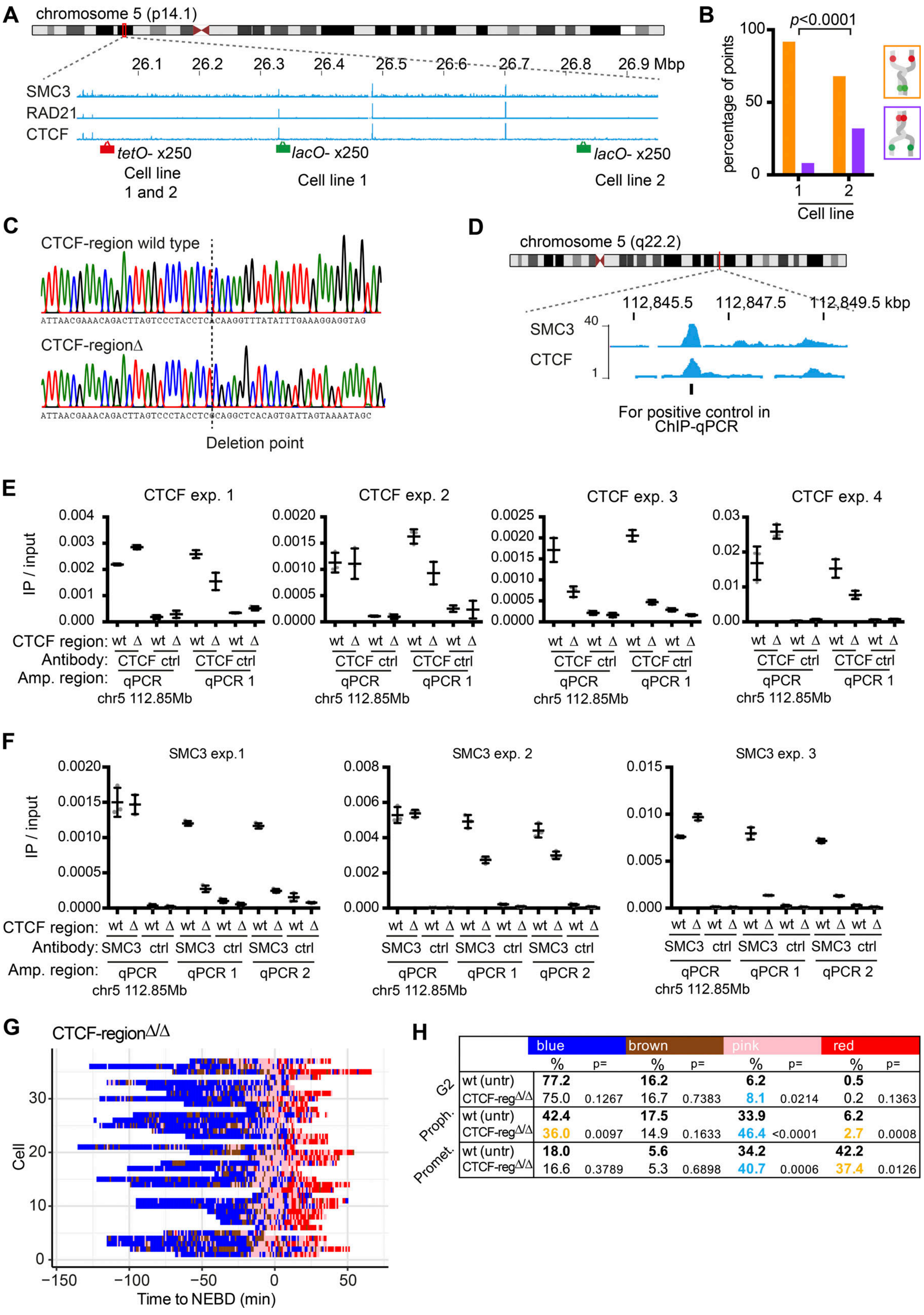

		blue %	p=	brown %	p=	pink %	p=	red %	p=
G2	wt (untr)	**77.2**		**16.2**		**6.2**		**0.5**	
	CTCF-regΔ/Δ	75.0	0.1267	16.7	0.7383	8.1	0.0214	0.2	0.1363
Proph.	wt (untr)	**42.4**		**17.5**		**33.9**		**6.2**	
	CTCF-regΔ/Δ	36.0	0.0097	14.9	0.1633	46.4	<0.0001	2.7	0.0008
Promet.	wt (untr)	**18.0**		**5.6**		**34.2**		**42.2**	
	CTCF-regΔ/Δ	16.6	0.3789	5.3	0.6898	40.7	0.0006	37.4	0.0126

Figure S3. **Deletion of CTCF-binding region and its outcome.** **(A)** The ChIP-seq data show the distribution of SMC3, RAD21, and CTCF along the genomic region where *tetO* and *lacO* were inserted in cell line 1 (TT75 and derivatives including TT104) and cell line 2 (TT68). The ChIP-seq data are taken from published data (ENCODE Project Consortium, 2012) and their GEO accession numbers are GSM935542 (SMC3), GSM935647 (RAD21), and GSM733645 (CTCF). **(B)** Graph shows the proportion of *tetO* (orange color) and *lacO* (purple color) sister separation among all "partial resolution" configurations in cell line 1 (TT104) and cell line 2 (TT68) during late G2 phase and prophase. In each case, microscopy images were taken by time lapse every 2 min. The P value was obtained using the chi-square statistical test, and the numbers of analyzed time points were 208 and 50 for cell line 1 and cell line 2, respectively. **(C)** Genome DNA sequencing of the CTCF-binding region from WT (TT75) and CTCF-region$^{\Delta/\Delta}$ (TT108) cells. Sequencing was performed across the gRNA1 region as shown in Fig. 4 C. The junction of the deletion is indicated by the dotted line. The CTCF-regionΔ shown here contains deletion of the 1277-bp region between gRNA 1 and gRNA 2 regions. **(D)** The ChIP-seq data show the distribution of SMC3 and CTCF along the genomic region where the qPCR positive control primers are situated. The ChIP-seq data are taken from published data (ENCODE Project Consortium, 2012) and their GEO accession numbers are GSM935542 (SMC3) and GSM733645 (CTCF). **(E and F)** Graphs show results of individual ChIP experiments followed by qPCR (ChIP-qPCR). An antibody against CTCF or SMC3, or a nonspecific antibody, was used for ChIP with WT (wt) or CTCF-region$^{\Delta/\Delta}$ (Δ) cells (TT75 and TT108, respectively). The genome intervals (qPCR1 and qPCR2 in Fig. 4 C) were amplified by PCR following ChIP. A region on chromosome 5 (112.85 Mbp) indicated by † in Fig. 4 B and zoomed in part D, where CTCF and cohesins are enriched, was also amplified by PCR as a control. In each ChIP experiment, three technical replicate PCRs were performed for each sample. From the individual qPCR amplification, the ratio of immunoprecipitated (IPed) DNA in ChIP to input DNA (IP/input) was determined. The bars and error bars on the graphs represent the mean and SD of technical replicates, respectively. Note that the variation among yield values between experiments may stem, for example, from different batches or freshness of antibodies and other reagents. Nonetheless, relative amounts among conditions showed consistent results between individual experiments. Therefore, normalized data from individual ChIP experiments (i.e., the mean of technical replicates from each ChIP experiment was normalized and taken as one data point) are shown in Fig. 4, D and E. **(G)** Behavior of the fluorescence reporter as observed in individual CTCF-region$^{\Delta/\Delta}$ live cells, plotted across the *y* axis. TT108 cells were arrested by a double-thymidine block and subsequently released from the block. 8–10 h after the release, images were taken every minute and, at each time point, the configuration of the reporter was determined and represented using the color codes depicted in Fig. 2, A and B. Data from individual cells were aligned relative to NEBD. White spaces represent time points where configuration could not be determined. **(H)** Table showing systematic analyses comparing the frequency of each conformation (nonresolved, blue; partially resolved, brown; resolved, pink; compacted, red) between WT or CTCF-region$^{\Delta/\Delta}$ cells during either G2 phase (−90 to −21 min), prophase (−20 to −1 min), and prometaphase (0 to 45 min). Percentages for the WT cells are shown in bold and black. The percentages obtained for the CTCF-region$^{\Delta/\Delta}$ cells are shown along with the P value calculated using a chi-square test. Where there is a statistically significant increase or decrease in frequency under the test conditions, the percentage is indicated in blue or orange, respectively. The data were taken from Fig. 2 C and Fig. S3 G. chr, chromosome; ctrl, control; exp, experiment; untr, untreated.

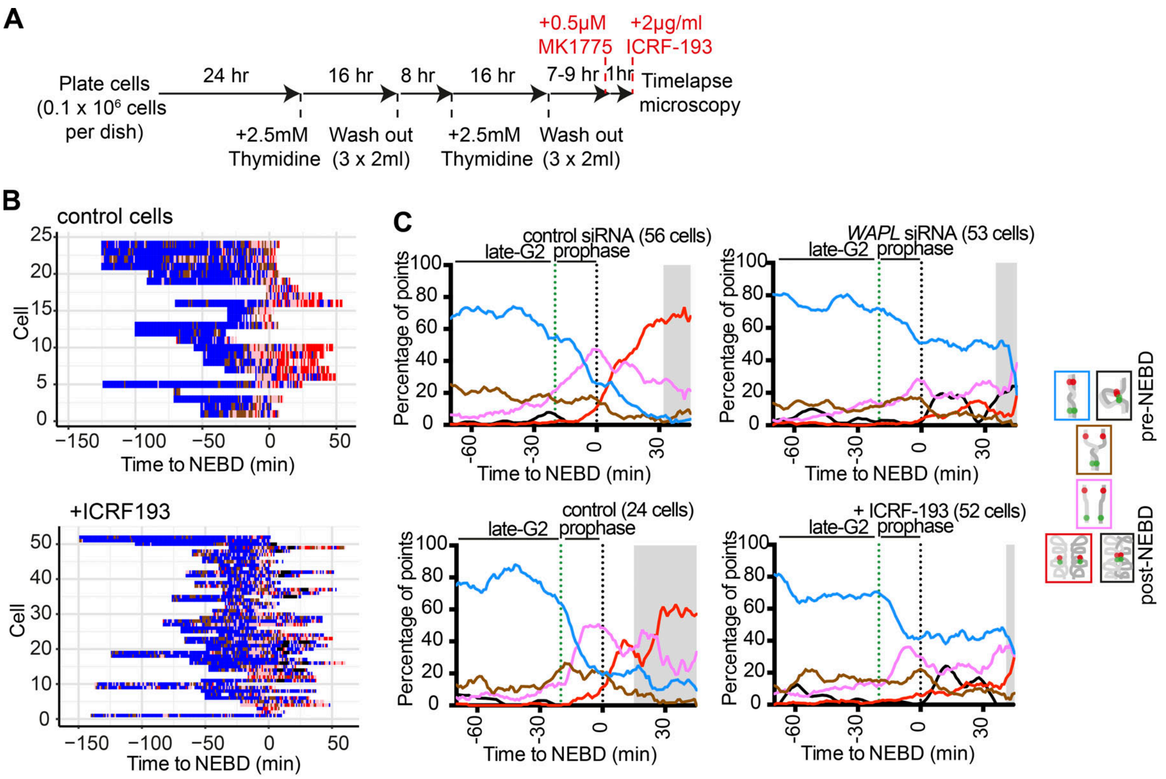

D

		blue %	blue p=	brown %	brown p=	pink %	pink p=	red %	red p=
G2	control	81.5		11.8		6.4		0.3	
	ICRF-193	73.2	<0.0001	14.9	0.0392	10.4	0.001	1.4	0.0103
Proph.	control	37.1		22.5		36.8		3.6	
	ICRF-193	54.4	<0.0001	15.3	0.007	27.4	0.0034	2.8	0.5338
Promet.	control	17.2		8.7		37.4		36.7	
	ICRF-193	54.3	<0.0001	11.3	0.1866	25.3	<0.0001	9.1	<0.0001

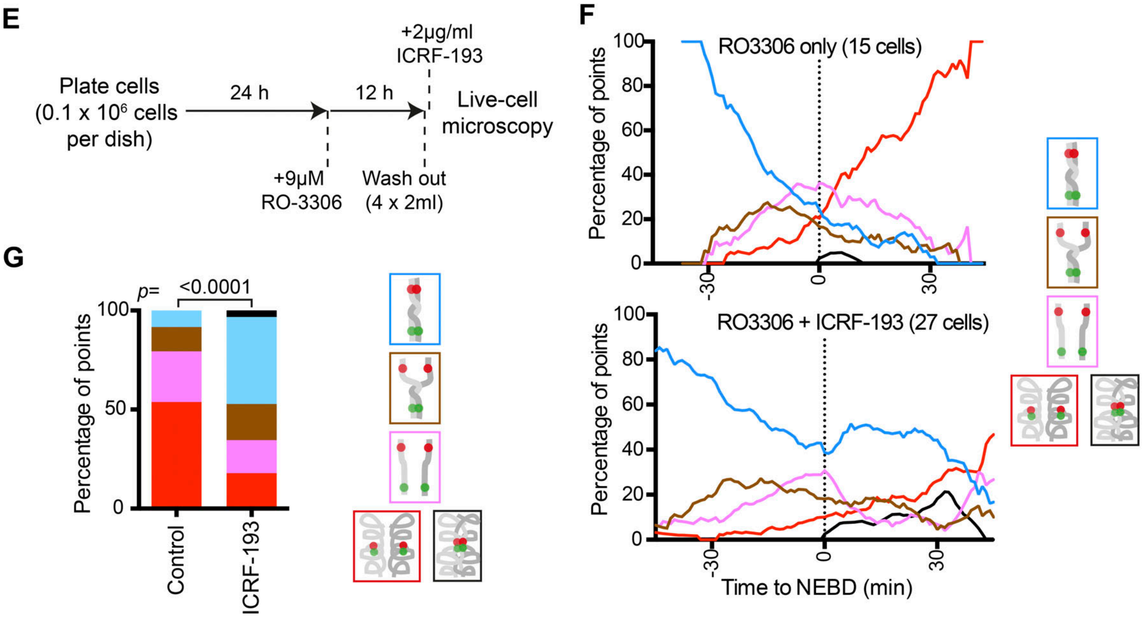

Figure S4. **Inhibition of topo II and its outcome. (A)** Experimental procedure outline. Cells were synchronized in early S phase using a double-thymidine block. Released cells were treated with MK1775 (WEE1 inhibitor) 1 h before and ICRF-193 (topo II inhibitor) 5–7 min before time-lapse imaging. **(B)** Behavior of the fluorescence reporter as observed in individual live cells, plotted across the *y* axis. TT75 cells were arrested by a double-thymidine block and subsequently released from the block. 8–10 h after the release cells were treated with MK1775 and ICRF-193 as described in A, images were taken every minute and, at each time point, the configuration of the reporter was determined and represented using the color codes depicted in Fig. 2, A and B. Data from individual cells were aligned relative to NEBD. White spaces represent time points where configuration could not be determined. **(C)** Evaluating specificity of the black state. With WAPL depletion and with ICRF-193 treatment, we often observed persistent (at least for five consecutive time points) colocalization of all four fluorescent dots, following NEBD (Fig. 3 E, right bottom; Fig. 6 B, right; and Fig. S4 F, bottom). We interpreted it as a "nonresolved and compacted" state and defined it as a black state. The black state was specific to WAPL depletion and ICRF-193 treatment, as it was very rarely observed in other conditions after NEBD. Next, we evaluated how often the configuration corresponding to the black state appeared before NEBD. For this, we applied the criteria for the black state (see Materials and methods) to the time points before NEBD, in the datasets for control siRNA, WAPL siRNA (Fig. 3 E), control treatment, and ICRF-193 treatment (Fig. 6 B). The configuration corresponding to the black state was observed in these conditions before NEBD, but only at a low percentage, as shown here. We interpret it as fortuitous continuous colocalization of red and green fluorescent dots (on nonresolved sister chromatids), since chromosome compaction is not substantial before NEBD (see the red "compacted" state in various conditions). Based on this interpretation, the configuration corresponding to the black state before NEBD was included in the blue "nonresolved" state in all analyses in figures, except for the figures shown here. Collectively, these data show that frequent appearance of black states (i.e., persistent colocalization of all four fluorescent dots) is specific to (a) WAPL depletion and ICRF-193 treatment and (b) post-NEBD. **(D)** Table shows systematic analyses comparing the frequency of each conformation (nonresolved, blue; partially resolved, brown; resolved, pink; compacted, red) between control (MK1775 treated) or ICRF-193 (with MK1775)–treated cells during either G2 phase (−90 to −21 min), prophase (−20 to −1 min), and prometaphase (0 to 45 min). Percentages for the control cells are shown in bold and black. The percentages obtained for the ICRF-193–treated cells are shown along with the P value calculated using a chi-square test. Where there is a statistically significant increase or decrease in frequency under the test conditions the percentage is indicated in blue or orange, respectively. The data were taken from Fig. S4 B. **(E)** Experimental procedure outline. Cells were arrested at the G2-M boundary by treatment with RO-3306 (CDK1 inhibitor). Cells were then released from the arrest and treated with ICRF-193 5–7 min before live-cell imaging. **(F)** Behavior of the fluorescence reporter observed in control cells or cells treated with ICRF-193. TT75 cells were synchronized as in E, and images were taken every minute, and at each time point, the configuration of the reporter was determined and represented using the color codes depicted on the right. Data from individual cells were aligned according to NEBD, and proportions of each configuration at each time point were determined with smoothing applied by calculating the rolling mean proportion across nine time points. **(G)** The change in fluorescence reporter configuration following NEBD was scored according to the pipeline shown in Fig. 6 E, left. The proportion of each state during the 20 min following NEBD for the selected cells is shown for control or ICRF-193–treated cells. P value was obtained by chi-square test, and the numbers of analyzed time points were 79 and 116 for control and ICRF-193–treated cells, respectively.

A

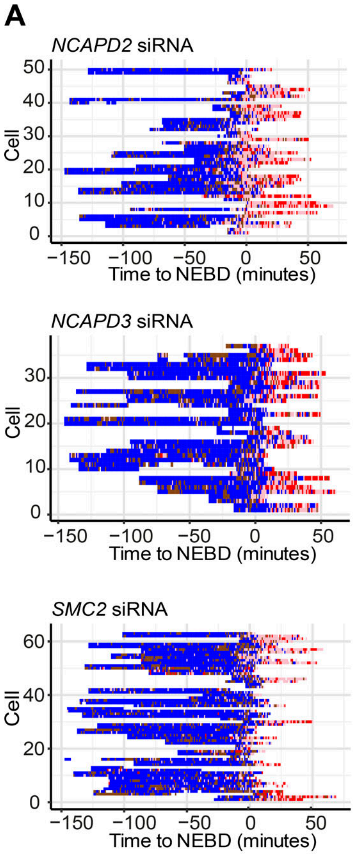

B

	siRNA	blue %	p=	brown %	p=	pink %	p=	red %	p=
G2	control	**71.3**		**19.6**		**8.5**		**0.6**	
	NCAPD2	82.2	<0.0001	12.2	<0.0001	5.0	<0.0001	0.6	0.9031
	NCAPD3	78.7	<0.0001	16.7	0.0341	4.1	<0.0001	0.5	0.5786
	SMC2	80.6	<0.0001	15.2	<0.0001	3.3	<0.0001	0.9	0.2467
Proph.	control	**46.5**		**15.4**		**34.0**		**4.1**	
	NCAPD2	44.3	0.3882	18.2	0.1267	32.8	0.5847	4.7	0.5636
	NCAPD3	71.1	<0.0001	18.2	0.1439	9.8	<0.0001	0.9	0.0001
	SMC2	56.8	0.0001	26.5	<0.0001	12.6	<0.0001	4.1	0.9686
Promet.	control	**15.4**		**7.7**		**35.6**		**41.2**	
	NCAPD2	10.5	0.0005	7.8	0.9152	56.9	<0.0001	24.8	<0.0001
	NCAPD3	31.5	<0.0001	8.3	0.6028	35.0	0.7619	25.2	<0.0001
	SMC2	24.1	<0.0001	14.1	<0.0001	44.5	<0.0001	17.4	<0.0001

C

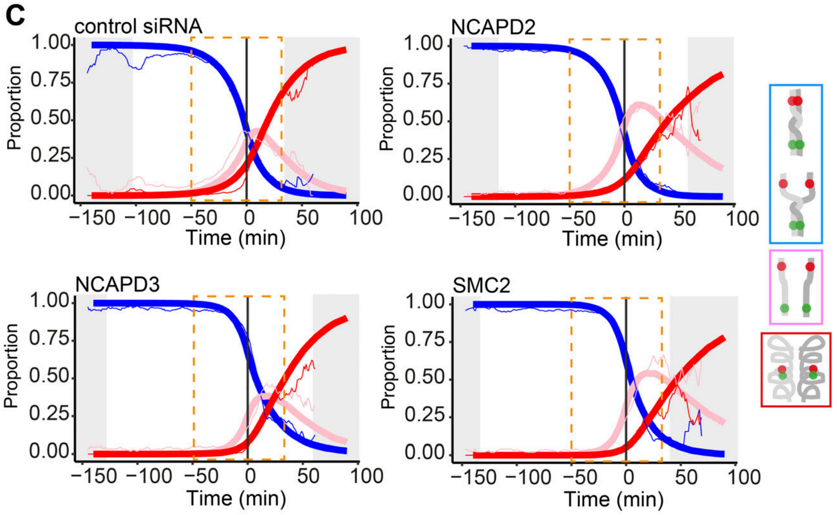

D

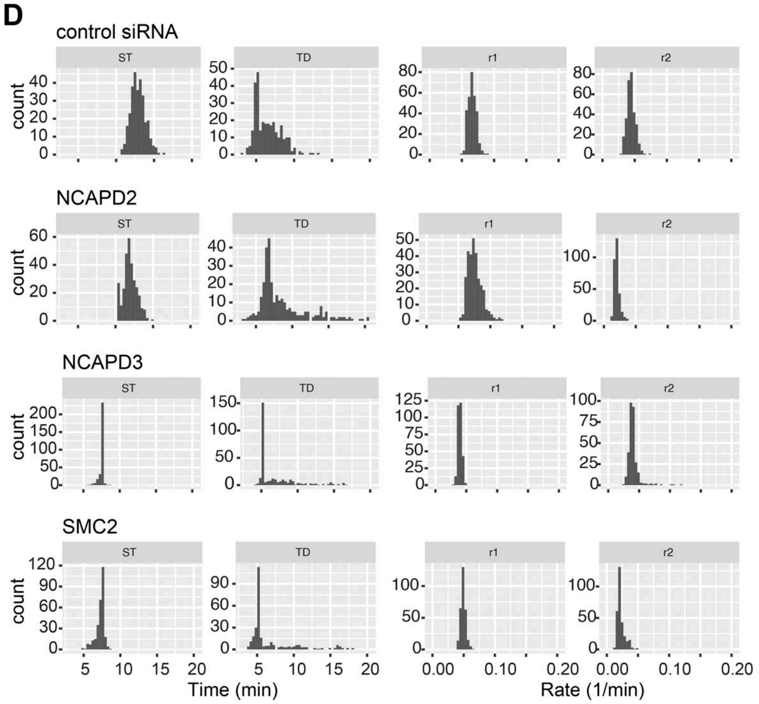

E

input: model parameters r_1, r_2, τ_1, τ_2,
number of cells m
time step Δt
output: proportion of state X at time t_k - $P(X; t_k)$

$p_1 \leftarrow 1 - e^{-r_1 \Delta t}$
$p_2 \leftarrow 1 - e^{-r_2 \Delta t}$
$T \leftarrow (-140, -139, \ldots, 90)$

for each i **in** $(1, 2, \ldots, m)$ **do**
  $\hat{t}_1 \leftarrow -rand.exp(1/\tau_1)$
  $\hat{t}_2 \leftarrow \hat{t}_1 + rand.exp(1/\tau_2)$
  $X \leftarrow$ "unresolved"
  for each t_k **in** T **do**
    $R \leftarrow rand.uni$
    if (X = "unresolved" **and** $t_k > \hat{t}_1$ **and** $R < p_1$)
      $X \leftarrow$ "resolved"
    else if(X = "resolved" **and** $t_k > \hat{t}_2$ **and** $R < p_2$)
      $X \leftarrow$ "compacted"
    fi
    $S_i(t_k) \leftarrow X$
  od
od

for each t_k **in** T **do**
  for each X **in** {"unresolved", "resolved", "compacted"} **do**
    $P(X;\, t_k) \leftarrow |\{i : S_i(t_k) = X\}| / m$
  od
od

Figure S5. **Depletion of condensin subunits and its outcome. (A)** Behavior of the fluorescence reporter as observed in individual live cells, treated with NCAPD2 (condensin I) or NCAPD3 (condensin II) or SMC2 (condensin I and II) siRNA, plotted across the *y* axis. TT75 cells were treated with siRNA, arrested by a double-thymidine block, and subsequently released from the block. 8–10 h after the release, images were taken every minute and, at each time point, the configuration of the reporter was determined and represented using the color codes depicted in Fig. 2, A and B. Data from individual cells were aligned relative to NEBD. White spaces represent time points where configuration could not be determined. **(B)** Table shows systematic analyses comparing the frequency of each conformation (nonresolved, blue; partially resolved, brown; resolved, pink; compacted, red) for each siRNA knockdown condition (NCAPD2, NCAPD3, or SMC2) with the frequency seen under control siRNA conditions during either G2 phase (−90 to −21 min), prophase (−20 to −1 min), and prometaphase (0 to 45 min). Percentages for the control conditions are shown in bold and black. The percentage for each test condition is shown along with the P value calculated using a chi-square test. Where there is a statistically significant increase or decrease in frequency under the test conditions the percentage is indicated in blue or orange, respectively. The data were taken from A, and control siRNA values are the same as in Fig. S2 L. **(C)** Microscopy data of cells treated with control, NCAPD2, NCAPD3, and SMC2 siRNA (thin lines) and their best-fitting models (thick lines). The lines represent the proportion of each configuration of the fluorescence reporter at each time point. The microscopy data lines were smoothed by a running mean with window size of 5 min, but model fitting was performed on the original data without smoothing. The color codes of lines are on the right. The gray-shaded area is as in Fig. 3 E. The orange-dotted box indicates the region where model fitting to microscopy data were performed. **(D)** Evaluating uncertainties of fit parameter values for TT75 cells treated with control, NCAPD2, NCAPD3, and SMC2 siRNA. Bootstrapping of microscopy data and subsequent model fitting were performed 300 times to create the distributions shown here (see more detail in Materials and methods). **(E)** Pseudocode describing the computer model used in Fig. 8. *rand.exp* and *rand.uni* refer to random number generators for exponential and uniform distribution, respectively.

References

ENCODE Project Consortium. 2012. An integrated encyclopedia of DNA elements in the human genome. *Nature.* 489:57–74. https://doi.org/10.1038/nature11247

Gibcus, J.H., K. Samejima, A. Goloborodko, I. Samejima, N. Naumova, J. Nuebler, M.T. Kanemaki, L. Xie, J.R. Paulson, W.C. Earnshaw, et al. 2018. A pathway for mitotic chromosome formation. *Science.* 359:eaao6135. https://doi.org/10.1126/science.aao6135

Liang, Z., D. Zickler, M. Prentiss, F.S. Chang, G. Witz, K. Maeshima, and N. Kleckner. 2015. Chromosomes progress to metaphase in multiple discrete steps via global compaction/expansion cycles. *Cell.* 161:1124–1137. https://doi.org/10.1016/j.cell.2015.04.030

Mora-Bermúdez, F., D. Gerlich, and J. Ellenberg. 2007. Maximal chromosome compaction occurs by axial shortening in anaphase and depends on Aurora kinase. *Nat. Cell Biol.* 9:822–831. https://doi.org/10.1038/ncb1606
